# Screening and identification of genes affecting grain quality and spikelet fertility during high-temperature treatment in grain filling stage of rice

**DOI:** 10.1186/s12870-021-03056-9

**Published:** 2021-06-07

**Authors:** Jae-Ryoung Park, Eun-Gyeong Kim, Yoon-Hee Jang, Kyung-Min Kim

**Affiliations:** 1 Division of Plant Biosciences, School of Applied Biosciences, College of Agriculture and Life Science, Kyungpook National University, Daegu, 41566 Republic of Korea; 2 Coastal Agriculture Research Institute, Kyungpook National University, Daegu, 41566 Republic of Korea

**Keywords:** Rice, High-temperature, Amylose, Grain quality, Spikelet fertility

## Abstract

**Background:**

Recent temperature increases due to rapid climate change have negatively affected rice yield and grain quality. Particularly, high temperatures during right after the flowering stage reduce spikelet fertility, while interfering with sugar energy transport, and cause severe damage to grain quality by forming chalkiness grains. The effect of high-temperature on spikelet fertility and grain quality during grain filling stage was evaluated using a double haploid line derived from another culture of F_1_ by crossing Cheongcheong and Nagdong cultivars. Quantitative trait locus (QTL) mapping identifies candidate genes significantly associated with spikelet fertility and grain quality at high temperatures.

**Results:**

Our analysis screened *OsSFq3* that contributes to spikelet fertility and grain quality at high-temperature. *OsSFq3* was fine-mapped in the region RM15749-RM15689 on chromosome 3, wherein four candidate genes related to the synthesis and decomposition of amylose, a starch component, were predicted. Four major candidate genes, including *OsSFq3*, and 10 different genes involved in the synthesis and decomposition of amylose and amylopectin, which are starch constituents, together with relative expression levels were analyzed. *OsSFq3* was highly expressed during the initial stage of high-temperature treatment. It exhibited high homology with FLOURY ENDOSPERM 6 in Gramineae plants and is therefore expected to function similarly.

**Conclusion:**

The QTL, major candidate genes, and *OsSFq3* identified herein could be effectively used in breeding rice varieties to improve grain quality, while tolerating high temperatures, to cope with climate changes. Furthermore, linked markers can aid in marker-assisted selection of high-quality and -yield rice varieties tolerant to high temperatures.

**Supplementary Information:**

The online version contains supplementary material available at 10.1186/s12870-021-03056-9.

## Background

Temperature affects rice yield and quality differently in each rice-growing stage [1–3]. High temperatures at early stage of rice growth have a positive effect. In particular, high-temperature stimulation at the beginning of growth promotes the absorption of nutrients to produce a lot of tiller, and can obtain high yield [4]. However, the high-temperature of the late growth period has a negative effect. In particular, the high-temperature of the grain filling stage increases the respiration volume of cells and reduces the accumulation of carbohydrates. In addition, by accelerating aging, the ripening period is shortened and the yield is reduced due to the decrease in the weight of the seed [5, 6]. In recent years, temperature increases and the frequency of unusually warm weather due to global warming are increasing, and these phenomena are known have a serious negative impact on the yield and quality of rice [7]. In the past 100 years, the average temperature of the earth has increased by 0.74 °C, and the rate of global warming has been increasing considerably [8]. According to IRRI, when the minimum temperature during the growing period of rice increases by 1 °C, the total rice yield decreases by > 10% [9]. In particular, during the entire growing period of rice, the reproductive stage is extremely sensitive to temperature [10], and the high-temperature stress during this period most severely affects fertility [11]. The high-temperature stress caused by global warming has a negative impact on all developmental stages, including all growth stages and yields of rice, and causes severe economic losses to rice farming worldwide [12]. Moreover, as winters decreased due to global warming and summers became relatively longer, the risk of exposure to high temperatures during the ripening of rice has increased. When rice is exposed to high temperatures during the right after the flowering stage, it causes widespread damage, such as a decrease in yield and quality deterioration due to a decrease in the fertility rate; hence, studies on high-temperature tolerance during the ripening stage of rice are highly important [13, 14]. Rice is most sensitive to temperature during flowering stage, and most sensitive to high temperatures before and immediately after flowering [15]. In particular, exposure to 38 °C for 1 h after the flowering period of rice dramatically increases the rate of infertility, and when exposed to 41 °C for 4 h, the rice suffers extremely serious damage, resulting in complete sterility [16, 17]. The optimum ripening temperature of Japonica rice is 21 °C–22 °C for 45 days after the flowering stage [18]. If the average temperature is > 26 °C at this time, the proportion of chalkiness in rice increases and the weight of grains also decreases. This phenomenon causes a decrease in yield [19, 20]. Primary cause of the occurrence of chalkiness rice is not the temperature in the early stage of ripening but the shortage of supply of assimilation products and a decrease in supply capacity after the middle stage of ripening. Moreover, the high-temperature stress during the ripening stage of rice is the major cause for the deterioration of the taste of rice by changing the activity of genes related to starch synthesis [21]. Although sugar is used as an energy source in plants, it has been shown to function as an important signaling material [22]. Furthermore, a large amount of sugar is introduced into the panicle during the ripening stage of rice, and the sugar signaling process is closely associated with the ripening process of rice grains [23]. High-temperature stress is one of the major environmental stresses that hinder grain development and growth by affecting sugar migration, which directly affects the quality and yield of seeds [24]. Not only high-temperature but also various abiotic stresses induce a common physiological response in the sugar/energy signaling system, thus indicating that an analysis of the sugar/energy signaling system and an analysis of the acting genes can play a vital role in the development of high-temperature-stress-tolerant rice varieties [25, 26]. Several studies have been conducted on the reduction of spikelet fertility in rice under high-temperature stress environment [27–29]. In particular, rice becomes infertile due to the lack of carbohydrates in the panicles at high temperatures, and changes in starch and starch biosynthetic enzyme activity in the ears at high temperatures also contribute to infertility [30]. At high temperatures, the accumulation of starch is suppressed through the metabolic processes of several transcripts in the early stages of rice grain filling, and the accumulation of amino acids increases, which results in a change in storage materials by high temperatures [31]. In particular, Boden et al., (2013) [32] reported that when high-temperature stress acts in the process of seed development in plants, there occurs a change in histone variant accumulation, and as the gene expression changes, the yield decreases. Currently, research on high-temperature-stress-tolerant rice is focused on increasing the survival rate and maintaining the yield under high-temperature conditions centered on heat shock proteins. However, as the process of forming rice grains is highly complex and several genes are involved, research on grain quality has been limited. Quantitative trait locus (QTL) has the ability to identify and manipulate genes associated with complex traits that regulate crop traits [33, 34]. QTL analysis provides rapid and accurate information on the genetic background by exploring specific components of the chromosome involved in the trait. Moreover, as differences in plant phenotypes are regulated by natural variation and environment, QTL analysis has the advantage of being able to identify the differences in phenotypes more practically than mutation analysis [35, 36]. Therefore, in this study, candidate genes related to spikelet fertility were identified under high-temperature treatment stress through QTL mapping, and among these candidate genes, genes that affect grain quality were screened. In addition, using the screened candidate genes, the relative gene expression and protein homology were identified in high-temperature-tolerant and -sensitive rice varieties.

## Results

### Rice grains ripened under high-temperature displayed a chalky appearance

**Fig. 1:**
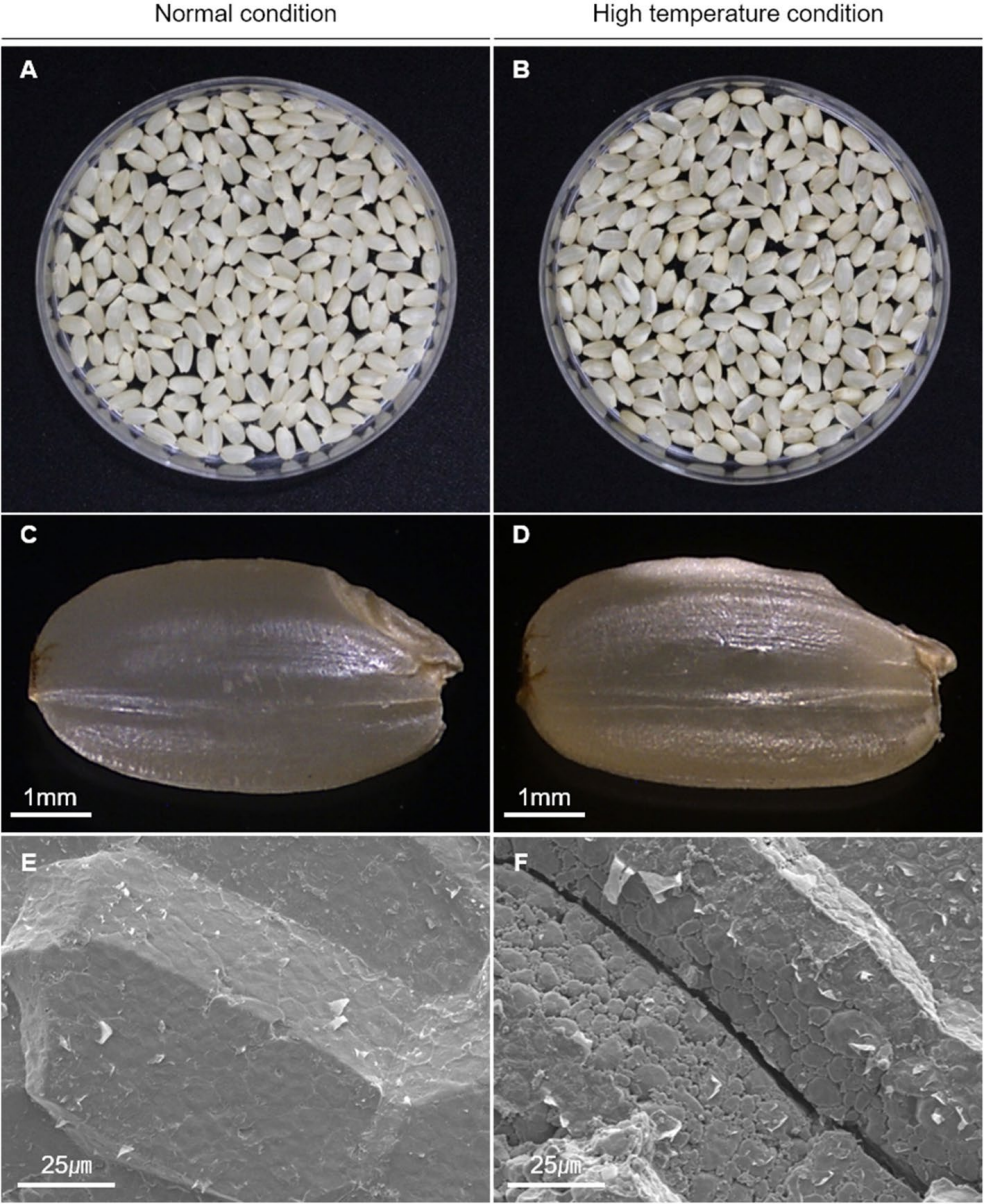
Appearance of grains exposed to control and high-temperature conditions. **A** and **B** Grain chalkiness was formed when grains were harvested after being exposed to high-temperature in the grain filling stage. The rate of grain chalkiness increases rapidly when exposed to high temperatures. The chalkiness grain negatively affects the grain quality. **C** and **D** When subjected to high-temperature, chalkiness grains are formed in which the middle portion of the seed turned white. Chalkiness grains have a low amylose content and a change in starch composition. **E** and **F** under a microscope, when subjected to high-temperature in the grain filling stage, a crack occurs in the starch structure

**Fig. 2:**
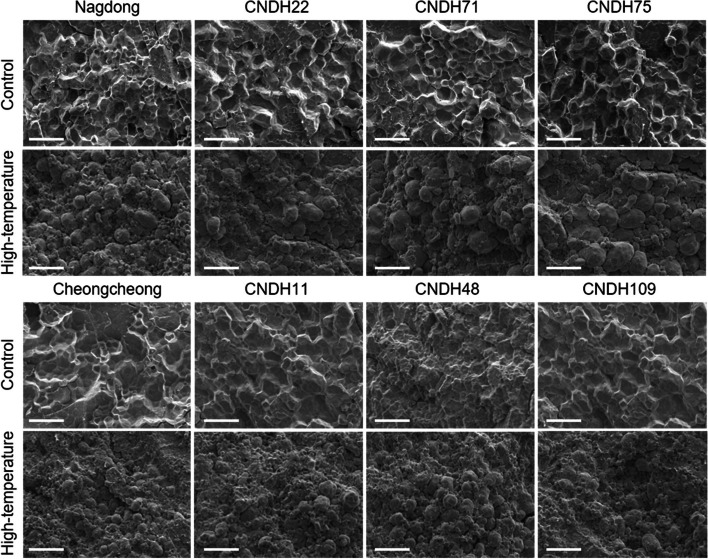
Scanning electron microscopy (SEM) analysis of the trnasverse sections of rice seed. Under normal conditions, amyloplasts are very robust and have a uniform shape. However, in high-temperature conditions, cracks occur between amyloplasts, and the shape changes to a round shape. This phenomenon occurs in both high-temperature tolerance lines and susceptible lines. However, high-temperature susceptible lines result in smaller amyloplast fragments. High-temperature-tolerant line; Nagdong, CNDH22, CNDH71, and CNDH75; high-temperature-susceptible line; Cheongcheong, CNDH11, CNDH48, and CNDH109

**Fig. 3:**
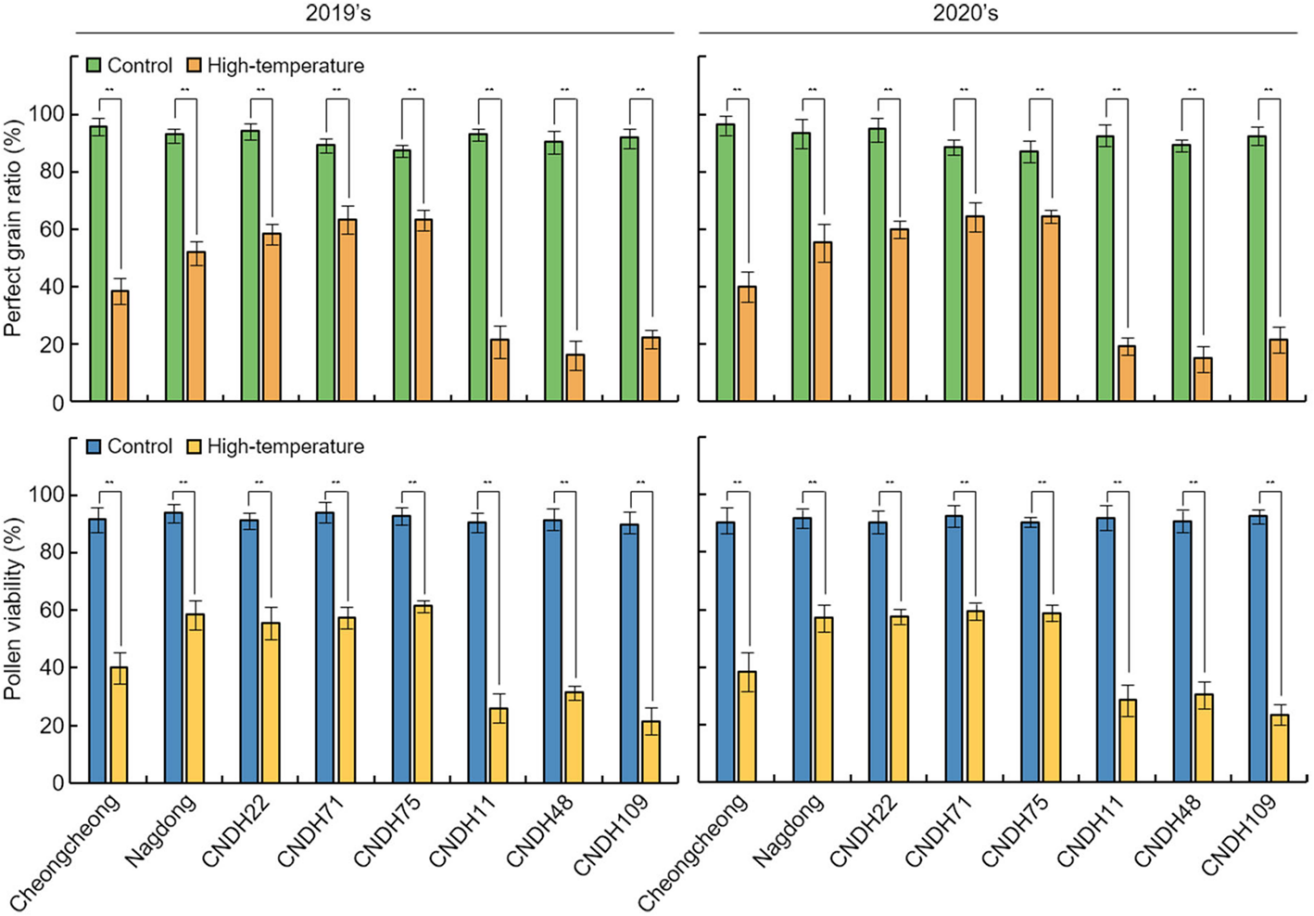
Comparison of high-temperature-tolerant and -sensitive lines for perfect grain ratio and pollen viability. When treated under high-temperature, perfect grain ratio and pollen viability decreased in both high-temperature-tolerant and -susceptible lines. However, the decrease in the ratio in the sensitive line was much higher than that in the high-temperature-tolerant line. High-temperature-tolerant line; Nagdong, CNDH22, CNDH71, and CNDH75; high-temperature-susceptible line; Cheongcheong, CNDH11, CNDH48, and CNDH109

When the rice was subjected to high-temperature treatment during the grain filling stage, the grains after ripening displayed a chalkiness appearance (Fig. 1). The middle portion of the grains changed to white due to high-temperature. However, the control samples had a perfect grain shape, mostly translucent, whereas the grains subjected to high-temperature stress exhibited an opaque appearance. When fully matured grains were analyzed by scanning electron microscopic, under normal conditions, amyloplasts were filled with polygonal shapes and tightly packed. However, in high-temperature conditioin, the shape was irregular, the amyloplasts were loose, and there were many gaps. In high-temperature conditions, the shape and arrangement of amyloplasts were changed, and thus, chalkiness grains were formed. This phenomenon was observed in both the high temperature tolerance line and the susceptible line. However, amyloplasts had more pores and irregular sizes in the susceptible line than in the high temperature tolerance line. In particular, CNDH22, CNDH71, and CNDH75 exhibited high-temperature tolerance among the CNDH 120 lines, and CNDH11, CNDH48, and CNDH109 were sensitive to high-temperature treatment (Fig. 2). For Cheongcheong, the perfect grain ratios were 95.7% in 2019 and 96.3% in 2020 under normal conditions, but when treated with high-temperature, the perfect grain ratios decreased to 38.4 and 40.2% in 2019 and 2020, respectively (Fig. 3). Nagdong had perfect grain ratios of 92.9% in 2019 and 93.4% in 2020 under normal conditions, but when treated with high-temperature, the perfect grain ratios decreased to 52.1 and 55.3% in 2019 and 2020, respectively. The ratio of perfect grain after high-temperature treatment was analyzed for the three lines CNDH22, CNDH71, and CNDH75, which showed tolerance to high-temperature among the CNDH 120 lines, and CNDH11, CNDH48, and CNDH109, which were sensitive to high-temperature. In 2019, the ratios of perfect grains of CNDH22, CNDH71, and CNDH75 were 94.3, 89.3, and 87.3%, respectively, under normal conditions, and 58.4, 63.5, and 63.4% under high-temperature conditions, respectively. In the high-temperature-tolerant CNDH line, the ratios of perfect grains of CNDH22, CNDH71, and CNDH75 that were reduced due to high-temperature were 38, 28, and27%, respectively. In 2020, the perfect grain ratios of CNDH22, CNDH71, and CNDH75 were 94.8, 88.7, and 87.2%, respectively, under normal conditions, and 60.1, 64.3, and 64.5%, respectively, under high-temperature conditions. In the high-temperature-tolerant CNDH line, the ratios of perfect grain reduced for CNDH22, CNDH71, and CNDH75 due to high-temperature were 36.6, 27.5, and 26.0%, respectively. We next analyzed the ratio of perfect grains under normal and high-temperature conditions for CNDH 11, CNDH48, and CNDH109, which were sensitive to high-temperature in the CNDH 120 line. In 2019, the perfect grain ratios of CNDH 11, CNDH48, and CNDH109 were 93.1, 90.5, and 91.8%, respectively, under normal conditions, and 21.4, 16.5, and 22.3%, respectively, under high-temperature conditions. In the high-temperature-sensitive CNDH line, the ratios of perfect grain reduction for CNDH11, CNDH48, and CNDH109 due to high-temperature were 77.0, 81.7, and 75.7%, respectively. In 2020, the ratios of perfect grains of CNDH11, CNDH48, and CNDH109 were 92.4, 89.3, and 92.5%, respectively, under normal conditions, and 19.3, 15.2, and 21.4% under high-temperature conditions, respectively. In the high-temperature-resistant CNDH line, the ratios of perfect grain reduction for CNDH11, CNDH48, and CNDH109 due to high-temperature were 79.1, 82.9, and 76.8%, respectively. Among the CNDH 120 lines, when both the tolerant lines and the sensitive line were treated with high-temperature, the perfect grain ratios decreased with a significant probability at the level of 1%.

### Changes in protein, amylose, and moisture content related to grain quality due to high-temperature treatment in grain filling stage

To analyze the effect of high-temperature during the filling stage of rice on protein, amylose, and moisture contents that affect grain quality, high-temperature was applied during the filling stage, and the contents of protein, amylose, and moisture were measured in the grains after harvesting (45 days after flowering) (Table 1). In 2019 and 2020, the protein contents of Cheongcheong were 6.1 ± 0.1% and 6.1 ± 0.1%, respectively, under normal conditions. However, the respective values under high-temperature conditions were 6.4 ± 0.1% and 6.3 ± 0.1%. Compared with the control group, the protein content of Cheongcheong increased with a significant probability at the 1% level under high-temperature conditions. In 2019 and 2020, the protein contents of Nagdong were 6.4 ± 0.3% and 6.5 ± 0.1%, respectively, under normal conditions. However, the respective values under high-temperature conditions were 6.7 ± 0.2% and 6.7 ± 0.1%. No significant difference was observed in the protein content of Nagdong compared with the control under high-temperature treatment during the filling stage for 2 years. Among the CNDH lines, differences in protein content were confirmed in CNDH22, CNDH71, and CNDH75, which were tolerant to high-temperature. All these three CNDH lines did not show any significant difference in protein content under both high-temperature treatment and normal conditions in 2019 and 2020. However, among the CNDH lines, CNDH11, CNDH48, and CNDH109, which were sensitive to high-temperature, showed increased protein content with a significant probability at 1% level in both 2019 and 2020 compared with the control under high-temperature treatment during the grain filling stage. Next, the amount of amylose that affects grain quality was measured in Cheongcheong, Nagdong, and CNDH lines. In 2019 and 2020, the amylose contents of Cheongcheong were 17.4 ± 0.1% and 17.3 ± 0.2%, respectively, under normal conditions. However, the respective values under high-temperature conditions were 15.1 ± 0.2% and 15.0 ± 0.1%. Compared with the control group, the amylose content of Cheongcheong decreased with a significant probability at the 1% level under high-temperature conditions. In 2019 and 2020, the amylose contents of Nagdong were 17.9 ± 0.1% and 17.9 ± 0.1%, respectively, under normal conditions. However, the values were 16.4 ± 0.1% and 16.4 ± 0.3% in 2019 and 2020 under high-temperature conditions, respectively. When Nagdong was subjected to high-temperature treatment during ripening for 2 years, the amylose content decreased with a significant probability at the 5% level compared to that in the control. Among the CNDH lines, differences in amylose content were confirmed in CNDH22, CNDH71, and CNDH75, which were highly resistant to high-temperature. When CNDH22 was subjected to high-temperature treatment during the filling stage, in 2019, the amylose content decreased with a significant probability at the 5% level compared to that in the control, but it decreased with a significant probability at the 1% level in 2020. Moreover, CNDH71 and CNDH75 showed a decreased amylose content with a significant probability at the 1% level in both 2019 and 2020. In addition, CNDH11, CNDH48, and CNDH109, which were sensitive to high-temperature among the CNDH lines, showed decreased amylose content with a significant probability at 1% level in both 2019 and 2020 compared to that in the control under high-temperature treatment during the filling stage. Finally, it was analyzed whether high-temperature treatment affects the moisture content of grains during the filling stage. Cheongcheong, Nagdong, CNDH22, CNDH71, and CNDH75, which have high-temperature tolerance capacity, and CNDH11, CNDH48, and CNDH109, which were sensitive, were all treated at high-temperature during the filling stage in 2019 and 2020. We observed no significant difference in the moisture content compared to that in the control.

**Table 1:** Changes in protein content, amylose content, and moisture content, which are major factors of grain quality, due to high-temperature treatment in the grain filling stage

CNDH population		Year	Protein content (%)			Amylose content (%)			Moisture content (%)		
			Control	High-temperature	*p* value	Control	High-temperature	*p* value	Control	High-temperature	*p* value
Parents	Cheongcheong	2019	6.1 ± 0.1^a^	6.4 ± 0.1	0.004^**^	17.4 ± 0.1	15.1 ± 0.2	0.002^**^	14.3 ± 0.1	14.1 ± 0.2	0.837
		2020	6.1 ± 0.1	6.3 ± 0.1	0.006^**^	17.3 ± 0.2	15.0 ± 0.1	0.004^**^	14.2 ± 0.1	14.1 ± 0.3	0.637
	Nagdong	2019	6.4 ± 0.3	6.7 ± 0.2	0.338	17.9 ± 0.1	16.4 ± 0.1	0.017^*^	14.9 ± 0.1	14.6 ± 0.3	0.150
		2020	6.5 ± 0.1	6.7 ± 0.1	0.245	17.9 ± 0.1	16.4 ± 0.3	0.031^*^	14.8 ± 0.2	14.5 ± 0.2	0.134
Tolerance	CNDH22	2019	6.4 ± 0.1	6.5 ± 0.2	0.384	18.1 ± 0.3	16.8 ± 0.1	0.028^*^	14.7 ± 0.1	14.6 ± 0.2	0.235
		2020	6.3 ± 0.2	6.5 ± 0.1	0.162	18.0 ± 0.1	16.7 ± 0.1	0.003^**^	14.8 ± 0.2	14.5 ± 0.3	0.388
	CNDH71	2019	6.3 ± 0.1	6.4 ± 0.1	0.547	18.1 ± 0.2	16.9 ± 0.3	0.002^**^	14.8 ± 0.2	14.6 ± 0.2	0.584
		2020	6.4 ± 0.3	6.5 ± 0.1	0.633	18.1 ± 0.3	17.0 ± 0.2	0.003^**^	14.8 ± 0.3	14.6 ± 0.2	0.132
	CNDH75	2019	6.3 ± 0.1	6.3 ± 0.2	0.248	18.3 ± 0.1	17.2 ± 0.1	0.004^**^	14.7 ± 0.2	14.5 ± 0.2	0.241
		2020	6.3 ± 0.1	6.2 ± 0.2	0.574	18.2 ± 0.1	17.2 ± 0.1	0.003^**^	14.5 ± 0.1	14.4 ± 0.3	0.754
Sensitive	CNDH11	2019	6.2 ± 0.3	7.1 ± 0.1	< 0.001^**^	17.3 ± 0.2	14.8 ± 0.2	< 0.001^**^	14.5 ± 0.2	14.3 ± 0.1	0.063
		2020	6.3 ± 0.2	7.0 ± 0.2	< 0.001^**^	17.3 ± 0.3	14.8 ± 0.1	< 0.001^**^	14.5 ± 0.3	14.3 ± 0.2	0.052
	CNDH48	2019	6.5 ± 0.1	7.3 ± 0.2	< 0.001^**^	17.8 ± 0.3	15.1 ± 0.1	< 0.001^**^	14.3 ± 0.2	14.1 ± 0.2	0.073
		2020	6.5 ± 0.1	7.3 ± 0.1	< 0.001^**^	17.7 ± 0.2	15.1 ± 0.3	< 0.001^**^	14.2 ± 0.2	14.2 ± 0.1	0.752
	CNDH109	2019	6.3 ± 0.3	7.1 ± 0.2	< 0.001^**^	18.2 ± 0.1	14.9 ± 0.2	< 0.001^**^	14.4 ± 0.1	14.1 ± 0.3	0.284
		2020	6.3 ± 0.2	7.1 ± 0.2	< 0.001^**^	18.1 ± 0.1	14.8 ± 0.2	< 0.001^**^	14.4 ± 0.1	14.0 ± 0.2	0.051

^a^Data are presented as mean ± standard deviation, *significant at the 0.05 level; **significant at the 0.01 level

### Effect of high-temperature in grain filling stage on pollen viability

**Fig. 4:**
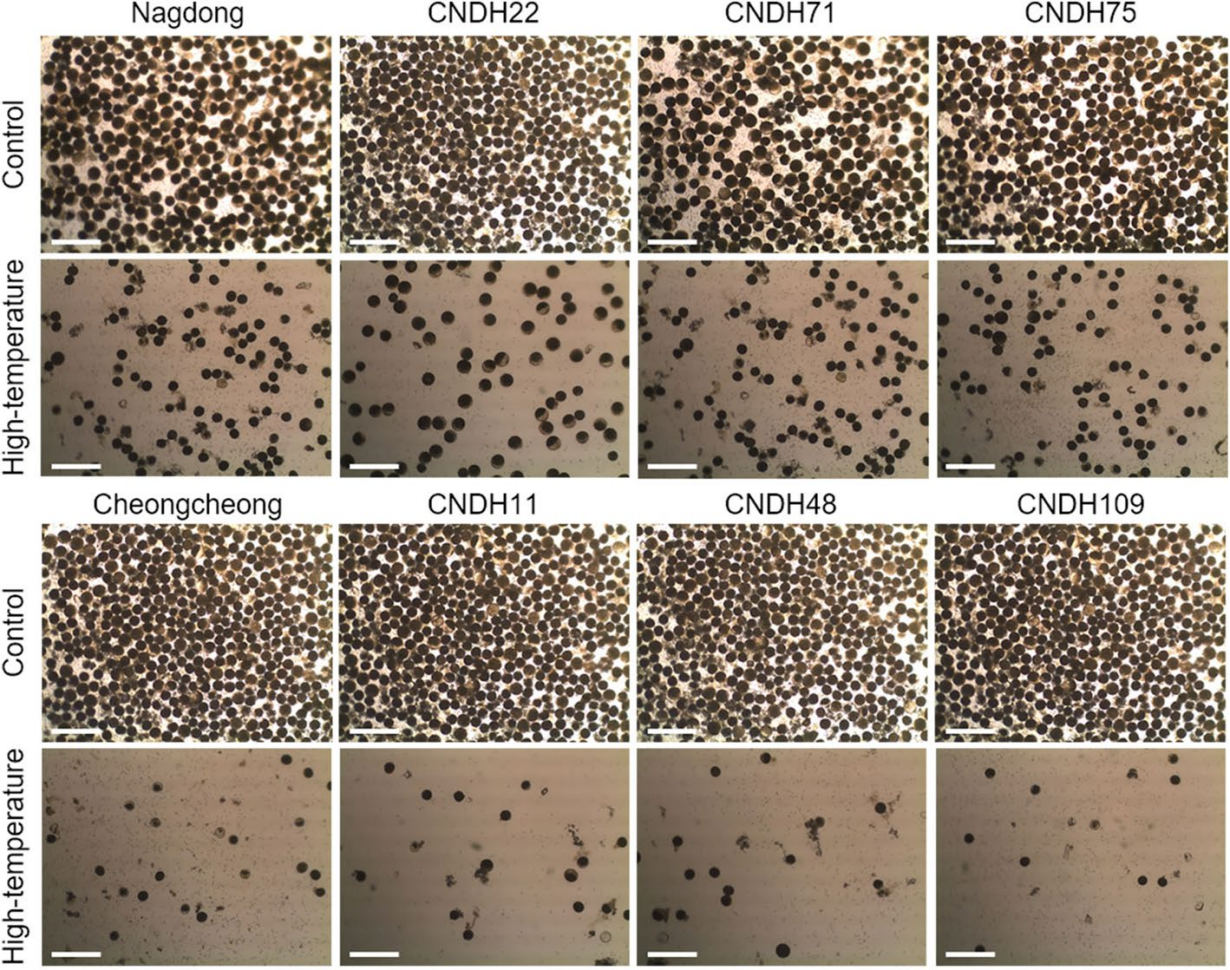
Effect of high-temperature on the pollen viability of rice under high-temperature stress in the flowering stage. When exposed to high temperatures during the flowering stage, the number of pollens decrease sharply. It also triggered decreases in the rate of spikelet fertility and yield. Pollen viability decreased in both tolerant line and sensitive line during high-temperature treatment, but decreased more sharply in the sensitive line. Pollen grains were stained using I_2_-KI. Nagdong, CNDH22, CNDH71, and CNDH75 were tolerant to high-temperature, and Cheongcheong, CNDH11, CNDH48, and CNDH109 were susceptible to high-temperature. Scale bars, 100 μm

Pollen is a key regulator of rice fertility. After high-temperature treatment immediately after flowering, pollen viability was investigated by I_2_-KI staining (Fig. 4). The average pollen viability was reduced in both the high-temperature-tolerant line and the high-temperature-sensitive line under high-temperature treatment immediately after flowering. In the control samples without high-temperature treatment, > 90% of pollen grains survived and showed a dark black and uniform round shape. However, under high-temperature treatment, the pollen grains did not have a uniform shape, and the shape varied. Moreover, the proportion of pollen grains of transparent color rather than dark black was high. The number of pollen grains also decreased. At high-temperature treatment in 2019, the pollen viability of Cheongcheong and Nagdong decreased by 56.1 and 37.7%, respectively, compared to that in the control. Under high-temperature treatment, CNDH22, CNDH71, and CNDH75 showed decreased pollen viability by 38.8, 38.6, and 33.7%, respectively, compared to that in the control, and CNDH11, CNDH48, and CNDH109 also showed decreased pollen viability by 71.6, 65.6, and 76.0%, respectively. High-temperature treatment in 2020 produced similar results as in 2019. Under high-temperature treatment, the pollen viability of Cheongcheong and Nagdong decreased by 57.4 and 37.7%, respectively, those of CNDH22, CNDH71, and CNDH75 decreased by 36.3, 35.7, and 34.9%, respectively, and those of CNDH11, CNDH48, and CNDH109 decreased by 68.8, 66.3, and 74.7%, respectively. When treated with high-temperature, the pollen viability decreased on average in both the high-temperature-tolerant and -sensitive lines, but the proportion of pollen viability in the susceptible line was significantly reduced compared to that in the tolerant line.

### Effect of high-temperature in grain filling stage on spikelet fertility and 1000 grain weight

**Fig. 5:**
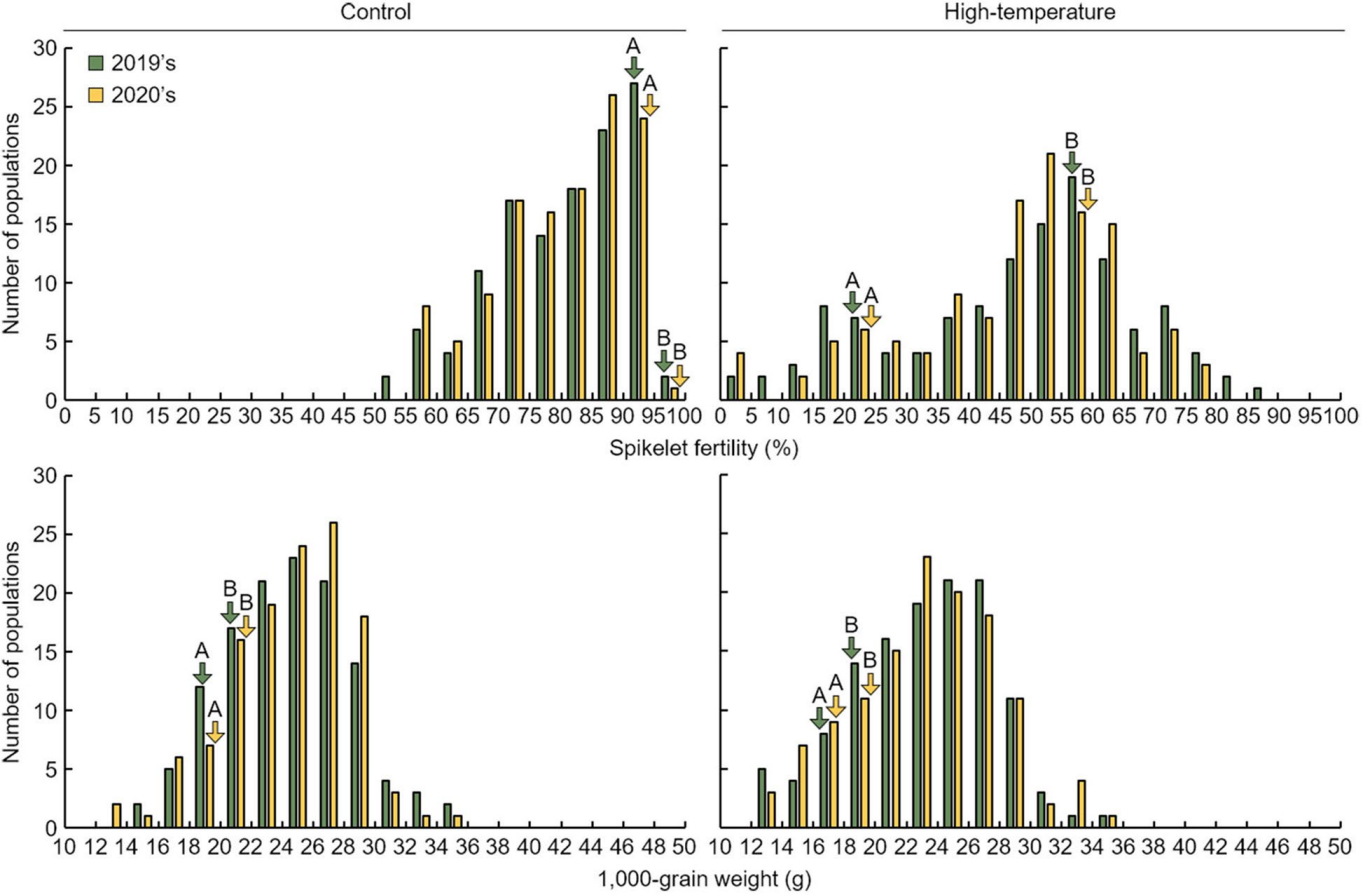
The frequency distribution for spikelet fertility and 1000 grain weight in the CNDH line. As spikelet fertility and 1000 grain weight showed a normal distribution, the spikelet fertility and 1000 grain weight are considered to be quantitative trait. This implies that various genes are involved in spikelet fertility and 1000 grain weight. A, Cheongcheong; B, Nagdong

To examine the effect of high-temperature on the grain formation process during the grain filling stage of rice, the spikelet fertility and 1000 grain weight were investigated in 2019 and 2020 (Table 2). The experiment was conducted by dividing the Cheongcheong, Nagdong, and CNDH 120 lines into two groups, normal condition and high-temperature condition of 42 °C. Under normal conditions, the average spikelet fertility values of Cheongcheong and Nagdong were 92.3 ± 1.3% and 97.8 ± 1.5% in 2019 and 93.5 ± 1.1% and 96.5 ± 1.7% in 2020, respectively. However, under high-temperature treatment, the spikelet fertility values of Cheongcheong and Nagdong decreased to 23.8 ± 2.1% and 53.8 ± 2.5% in 2019 and to 24.7 ± 1.8% and 58.6 ± 2.3% in 2020, respectively. All these values decreased with a significant probability at the 1% level in the experimental group compared with the control group. Under normal conditions in the CNDH 120 line, the average spikelet fertility values were 81.4 ± 11.3% in 2019 and 81.5 ± 10.5% in 2020, but under high-temperature conditions, they were 47.6 ± 19.4% in 2019 and 46.6 ± 17.2% in 2020. The CNDH 120 line also showed decreased values for 2 consecutive years with a significant probability at the 1% level under high-temperature conditions. Regarding the effect of high-temperature stress on the 1000 grain weight during the grain filling stage, under normal conditions, the 1000 grain weight of Cheongcheong was 19.3 ± 1.4 g in 2019 and 19.1 ± 1.2 g in 2020, and that of Nagdong was 21.8 ± 1.1 g in 2019 and 21.5 ± 1.2 g in 2020. The 1000 grain weight of the CNDH 120 line was 22.7 ± 4.2 g in 2019 and 22.9 ± 3.9 g in 2020. However, under high-temperature conditions, the 1000 grain weight of Cheongcheong was 16.2 ± 0.8 g in 2019 and 16.5 ± 0.7 g in 2020, and that of Nagdong was 18.2 ± 0.9 g in 2019 and 18.5 ± 0.8 g in 2020. The average of the 1000 grain weight of the CNDH 120 line was 20.8 ± 4.5 g in 2019 and 21.0 ± 4.6 g in 2020. The 1000 grain weights of Cheongcheong, Nagdong, and CNDH 120 lines all decreased under high-temperature conditions for 2 consecutive years, and these decreases were significant with a probability at the 1% level. When the Cheongcheong, Nagdong, and CNDH 120 lines were subjected to normal and high-temperature treatment conditions, the frequency distribution of the spikelet fertility and 1000 grain weight showed a continuous variation almost similar to the normal distribution. Therefore, the spikelet fertility and 1000 grain weight were related to more than one gene. This implies that the genes related to spikelet fertility and 1000 grain weight are a quantitative trait (Fig. 5).

**Table 2:** Spikelet fertility and 1000 grain weight of 120 Cheongcheong/Nagdong double haploid (CNDH) population

Plant Traits	Year	Parents						DH population		
		Cheongcheong			Nagdong					
		Control	High-temperature	*p* value	Control	High-temperature	*p* value	Control	High-temperature	*p* value
Spikelet fertility (%)	2019	92.3 ± 1.3^a^	23.8 ± 2.1	< 0.001^**^	97.8 ± 1.5	53.8 ± 2.5	< 0.001^**^	81.4 ± 11.3	47.6 ± 19.4	< 0.001^**^
	2020	93.5 ± 1.1	24.7 ± 1.8	< 0.001^**^	96.5 ± 1.7	58.6 ± 2.3	< 0.001^**^	81.5 ± 10.5	46.6 ± 17.2	< 0.001^**^
1000 grain weight (g)	2019	19.3 ± 1.4	16.2 ± 0.8	< 0.001^**^	21.8 ± 1.1	18.2 ± 0.9	< 0.001^**^	22.7 ± 4.2	20.8 ± 4.5	0.001^**^
	2020	19.1 ± 1.2	16.5 ± 0.7	< 0.001^**^	21.5 ± 1.2	18.5 ± 0.8	< 0.001^**^	22.9 ± 3.9	21.0 ± 4.6	0.001^**^

^a^Data are presented as mean ± standard deviation, *significant at the 0.05 level; **significant at the 0.01 level

### Analysis of QTLs associated with spikelet fertility and 1000 grain weight at high-temperature

**Fig. 6:**
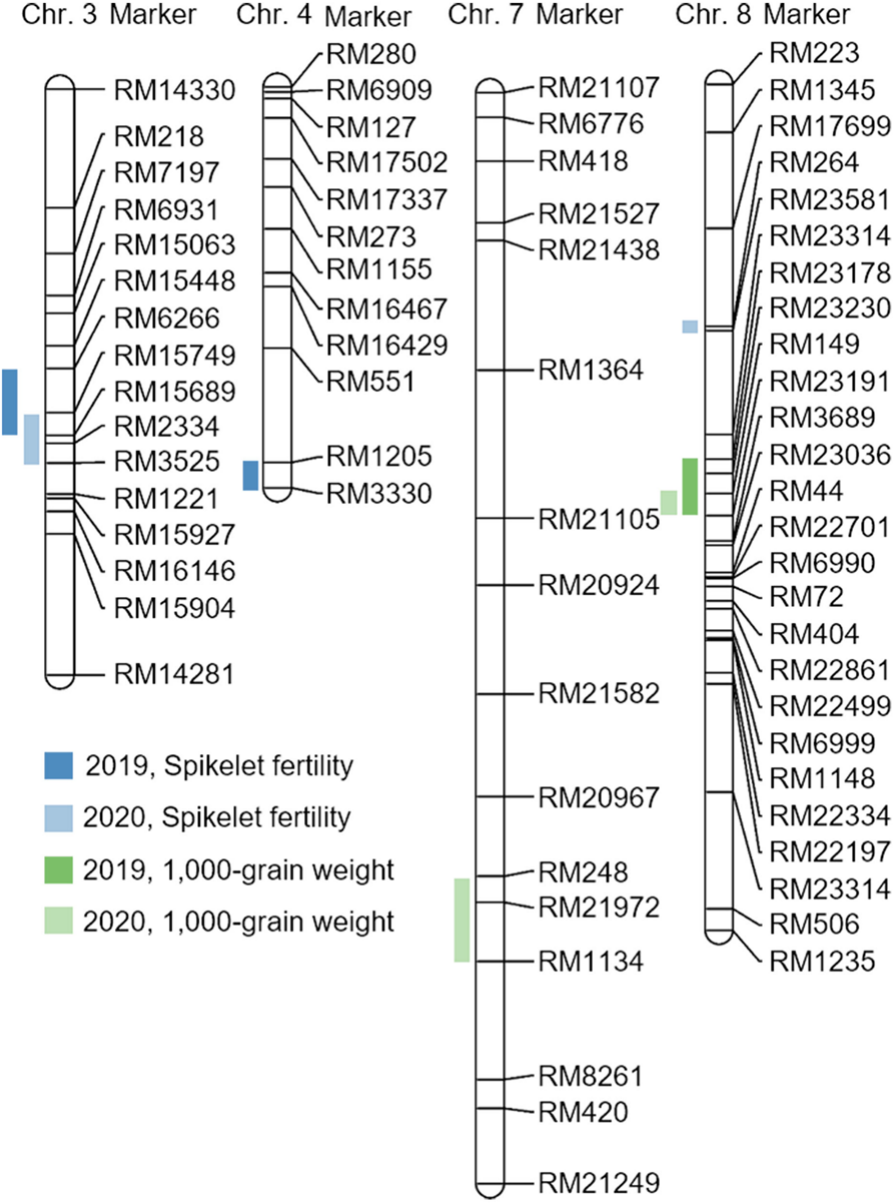
The chromosomal location of QTLs associated with spikelet fertility and 1000 grain weight. The QTLs mapped in chromosomes 3, 4, 7, and 8. Spikelet fertility-related QTL was mapped in RM15749-RM15689 on chromosome 3 for 2 consecutive years. The QTLs related to 1000 grain weight were detected in the same region for 2 years in RM149-RM23191 on chromosome 8

**Fig. 7:**
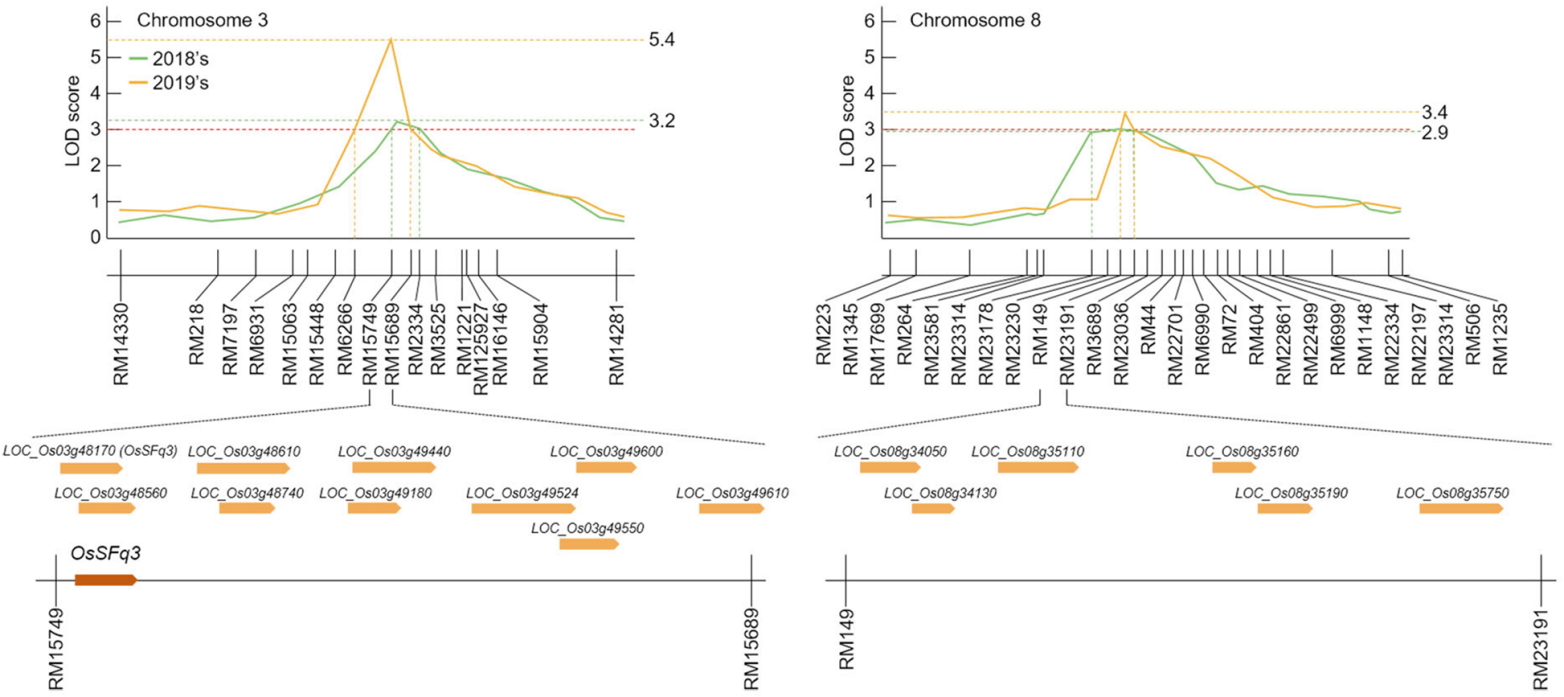
Physical map related to spikelet fertility and 1000 grain weight in rice. On chromosomes 3 and 8, an LOD score of ≥3.0 for 2 consecutive years was analyzed. Candidate genes related to sucrose energy were screened around marker regions, which were detected for 2 consecutive years, and *OsSFq3* was detected on chromosome 3. *OsSFq3* is a glycosidase-like gene that plays a key role in the transfer of glucose energy and affects spikelet fertility and grain quality

Genetic maps of the CNDH line were generated using 788 SSR markers. According to the results of polymorphism analysis for Cheongcheong and Nagdong, 423 SSR markers exhibited polymorphism. Among these 423 SSR markers selected through polymorphism analysis, 143 SSR markers, which represent codominants, and where PCR amplification occurs in both Cheongcheong and Nagdong, were selected and used to generate a genetic map of the CNDH line. The total length of the association map of the CNDH line was 2121.7 cM, and the average distance between the markers used to generate the genetic map was 10.6 cM. There were 19–50 markers per chromosome used in the CNDH line genetic map, and all these SSR markers were evenly distributed on 12 chromosomes in rice. High-temperature treatment was performed on Cheongcheong, Nagdong, and CNDH 120 lines during the grain filling stage, and of QTL mapping analysis was conducted using the CIM method of Windows QTL cartographer 2.5 on the spikelet fertility, 1000 grain weight, and genotype information that appeared after high-temperature treatment (Fig. 6). As a result of QTL mapping, which is involved in spikelet fertility and 1000 grain weight when high-temperature treatment during grain filling stage, qSf3 was detected on chromosome 3, qSf4 was detected on chromosome 4, and qTgw8 was detected on chromosome 8 in 2019. In 2020, qSf3–1 was detected on chromosome 3, qTgw7 was detected on chromosome 7, and qSf8 and qTgw8–1 were detected on chromosome 8. Among these, qSf3, qSf3–1, qSf4, and qSf8 are QTLs related to the fertility rate under high-temperature treatment during the grain filling stage, and qTgw7, qTgw8, and qTgw8–1 are QTLs related to the 1000 grain weight under high-temperature treatment during the grain filling stage. In 2019, qSf3 and qSf4 were explored for QTLs related to the spikelet fertility under high-temperature treatment during the grain filling stage. qSf3 was detected in the RM15749-RM2334 region of chromosome 3, and the LOD score was 3.2. The phenotypic variation that can be explained was 20%, derived from the allele of Cheongcheong. qSf4 was detected in the RM1205-RM3330 region of chromosome 4, and the LOD score was 2.8. The explainable phenotypic variation was 40%, derived from the Cheongcheong allele. In 2020, qSf3–1 and qSf8 were explored for QTLs related to the spikelet fertility under high-temperature treatment during the grain filling stage. qSf3–1 was detected in the RM6266-RM15689 region of chromosome 3, and the LOD score was 5.4. The phenotypic variation that can be explained was 10%, derived from the Cheongcheong allele. qSf8 was detected in the RM264-RM23581 region of chromosome 8, and the LOD score was 3.1. The descriptive phenotypic variation was 30%, derived from the Cheongcheong allele. RM15749-RM15689 of chromosome 3 was commonly detected in 2019 and 2020 when QTL mapping was performed on the spikelet fertility under high-temperature treatment during the grain filling stage of rice, all of which had an LOD score of ≥3.0 and were derived from the Cheongcheong allele. In 2019, qTgw8 was detected for QTL related to the 1000 grain weight under high-temperature treatment during the grain filling stage. qTgw8 was detected in the RM23178-RM23191 region of chromosome 8, and the LOD score was 3.1. The phenotypic variation that can be explained was 20%, derived from the allele of Nagdong. In 2020, qTgw7 and qTgw8–1 were detected for QTLs related to the 1000 grain weight under high-temperature treatment during the grain filling stage. qTgw7 was detected in the RM248-RM1134 region of chromosome 7, and the LOD score was 2.7. The phenotypic variation that can be explained was 20%, derived from the allele of Cheongcheong. qTgw8–1 was detected in the RM149-RM23191 region of chromosome 8, and the LOD score was 3.4. The phenotypic variation that can be explained was 10%, derived from the allele of Nagdong. RM149-RM23191 of chromosome 8 was commonly detected in 2019 and 2020 during QTL mapping related to the 1000 grain weight under high-temperature treatment during the grain filling stage, and the LOD scores of this region were 3.1 and 3.4, respectively. It was derived from the Nagdong allele (see Additional file 1: Table S1 and Fig. 7).

### Search for candidate genes associated with grain quality and spikelet fertility based on QTL mapping

**Fig. 8:**
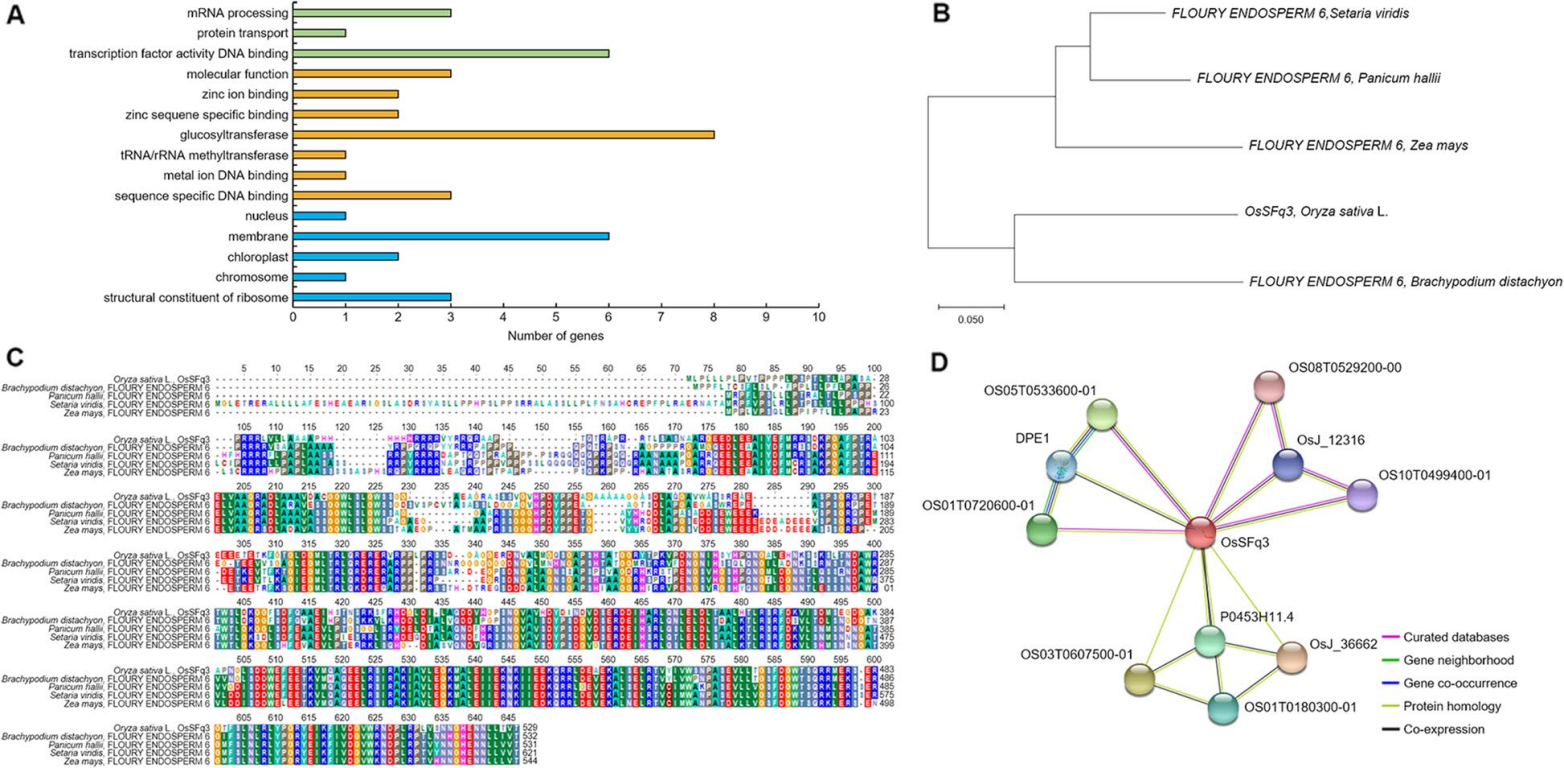
Predicting the function of OsSFq3. **A** Significantly enriched candidate genes located in RM15749-RM15689 on chromosome 3 and RM149-RM13191 on chromosome 8. The green box represents biological processes, the yellow box represents molecular function, and the blue box represents cellular components. **B** Genetic homology analysis of *OsSFq3* and Gramineae plants through phylogenetic tree. The phylogenetic tree was constructed by the parsimony method with 1000 bootstrap replicates. **C** The domain of *OsSFq3* has very high homology with the FLOURY ENDOSPERM protein and is therefore predicted to have a similar function. **D** Protein interaction of OsSFq3. OsSF3 interacts with OS08T0529200–00, OsJ_12316, OS10T0499400–01, OS05T0533600–01, DPE1, OS01T0720600–01, P0453H11.4, OS03T0607500–01, OS01T0180300–01, and OsJ_36662. All these are involved in the synthesis of starch, and high-temperature treatment in the grain filling stage changes the composition of the starch by controlling these proteins

In 2019 and 2020, the results of QTL mapping related to spikelet fertility and 1000 grain weight under high-temperature treatment during the grain filling stage of rice detected two QTLs on chromosome 3, one QTL on chromosome 4, one QTL on chromosome 7, and three QTLs on chromosome 8. Among them, RM15749-RM15689 of chromosome 3 is a region commonly detected for 2 years related to spikelet fertility, and RM149-RM23191 of chromosome 8 is also a region commonly detected in 2019 and 2020. The marker interval of RM15749-RM15689 of chromosome 3 was 7.1 cM, and the marker interval of RM149-RM23191 of chromosome 8 was 7.0 cM. These SSR markers were analyzed by NCBI, and a total of 34 high-temperature-related candidate genes were searched. They were classified according to their function. RM15749-RM15689 on chromosome 3 and RM149-RM23191 on chromosome 8 included ORFs involved in biological processes, ORFs involved in molecular functions, and ORFs involved in cellular components (see Additional file 1: Table S2). The candidate genes involved in biological processes consisted of mRNA processing, protein transport, and transcription factor activity DNA-binding proteins (Fig. 8). The candidate genes involved in molecular functions consisted of zinc ion binding, zinc sequence-specific binding, glucosyltransferase, tRNA/rRNA methyltransferase, metal ion DNA-binding, and sequence-specific DNA-binding proteins. The candidate genes involved in cellular components consisted of nuclear, membrane, chloroplast, chromosome, and structural constituent of ribosome proteins. Among them, *LOC_Os03g48170*, located on chromosome 3, is a glycoside hydrolase, family 13, and N-terminal domain-containing protein, and *LOC_Os03g49600* and *LOC_Os03g49610* are genes with a sequence similar to that of beta-glucosidase. *LOC_Os08g35110*, located on chromosome 8, is an auxin-responsive SAUR protein family protein. All these genes are related to the synthesis and decomposition of starch and sugar energy transfer. Therefore, *LOC_Os03g48170*, *LOC_Os03g49600*, *LOC_Os03g49610*, and *LOC_Os08g35110* were screened as candidate genes related to spikelet fertility and grain quality under high-temperature treatment during the grain filling stage.

### Candidate gene expression levels in grain filling stage under high-temperature treatment

**Fig. 9:**
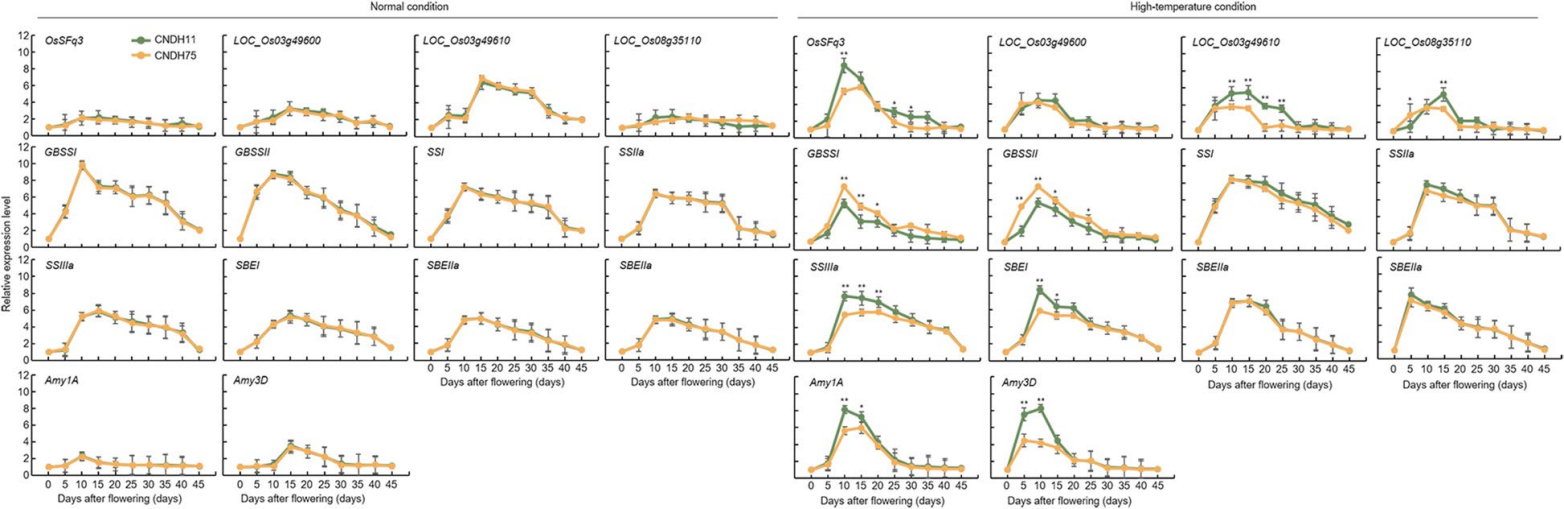
Analysis of relative expression levels of candidate genes and starch synthesis or degrading enzymes in high-temperature-sensitive and -tolerant lines under high-temperature stress and normal conditions. Analysis of candidate gene expression of rice in the flowering stage exposed to 42 °C in the growth chamber and sampling at various time points of 0, 5, 10, 15, 20, 25, 30, 35, 40, and 45 days after flowering. There was no significant difference in all genes under normal conditions, but there were significant differences in some genes in the high-temperature-tolerant and -sensitive lines under high-temperature treatment. CNDH11 is a high-temperature-sensitive line, and CNDH75 is a high-temperature-resistant line. * Significantly different at the 0.05 level. ** Significantly different at the 0.01 level

GBSSI, GBSSII, SSI, SSIIa, SSIIIa, SBEI, SBEIIa, SBEIIb, Amy1A, and Amy3D, which are involved in the synthesis and decomposition of starch from seeds during the ripening of rice and candidate genes related to the spikelet fertility of high-temperature screening through this research such as LOC_Os03g48170, LOC_Os03g49600, LOC_Os03g49610, and LOC_Os08g35110 compared relative expression level (Fig. 9). High-temperature treatment was conducted at the beginning of flowering, and panicles were sampled every 5 days. The grains were harvested 45 days after flowering and sampled at a total of nine times. Under normal conditions without high-temperature stress, there was no significant difference in the relative expression levels of genes involved in starch degradation and synthesis, including *LOC_Os03g48170*, *LOC_Os03g49600*, *LOC_Os03g49610*, and *LOC_Os08g35110* in CNDH11 and CNDH75. In addition, all the genes involved in starch synthesis exhibited the highest expression level on the 10th or 15th day after flowering. However, when the high-temperature treatment was started during the grain filling stage, there was a difference in the relative expression levels of genes involved in starch synthesis and degradation in CNDH11 and CNDH75. The relative expression levels of the amylose synthesis genes *GBSSI* and *GBSSII*, which are components of starch, were decreased in both CNDH11 and CNDH75. However, there was a high rate of decrease in CNDH11, which has a high-temperature sensitivity, compared with CNDH75, which has a high-temperature tolerance, and these decreases were significant at the 1% level. SSI, SSIIa, SSIIIa, SBEI, SBEIIa, and SBEIIb are amylopectin synthesis genes, which are constituents of starch. SSI, SSIIa, SBEIIa, and SBEIIb did not show a significant difference in the amount of relative gene expression level compared with control when subjected to high-temperature treatment. When SSIIIa was treated with high-temperature, its expression was increased in both CNDH11 and CNDH75. However, the rate of increase in CNDH11 was higher, and this difference was significant at the 1% level on the 10th, 15th, and 20th days after flowering. SBEI showed increased relative expression level in both CNDH11 and CNDH75 when treated with high-temperature. However, the rate of increase in CNDH11 was higher, and these increases were significant at the 1% level at 10 days after flowering and at the 5% level at 15 days after flowering. After the high-temperature treatment in the grain filling stage, the relative expression levels of the amylose decomposing enzymes Amy1A and Amy3D were identified. The relative expression levels of these enzymes were increased compared to those in the control when subjected to high-temperature treatment, and the rate of increase in CNDH11 was higher than that in CNDH75. Moreover, Amy1A expression level increased with a significant difference at the 1% level after 10 days of flowering and further increased with a significant difference at the 5% level on the 15th day after flowering. Amy3D expression level also increased with a significant difference at the 1% level on the 5th and 10th days after flowering. The relative expression levels of genes related to spikelet fertility and grain quality under high-temperature treatment, viz., *LOC_Os03g48170*, *LOC_Os03g49600*, *LOC_Os03g49610*, and *LOC_Os08g35110* screened through QTL mapping, were checked. Under high-temperature treatment in the grain filling stage, compared with the control, the expression of *LOC_Os03g48170* was increased with a significant difference at the 1% level after 10 days of flowering. There was no significant difference in the relative expression level of *LOC_Os03g49600* under high-temperature and control conditions. When *LOC_Os03g49610* was subjected to high-temperature, its relative expression level was decreased with a significant difference at the 1% level at 10, 15, 20, and 25 days after flowering compared to that in the control. Compared with the control, the relative expression level of *LOC_Os08g35110* increased with a significant difference at the 5% level on the 5th day after flowering, and it further increased with a significant difference at the 1% level on the 15th day after flowering.

### Analysis of phylogenetic tree and homology sequence

The candidate genes *LOC_Os03g48170*, *LOC_Os03g49600*, *LOC_Os03g49610*, and *LOC_Os08g35110* that affect spikelet fertility and grain quality were screened under high-temperature treatment during the grain filling stage of rice. In addition, when the candidate gene relative expression levels were analyzed, the gene expression patterns of *LOC_Os03g48170* were similar to those of the amylose-degrading enzymes Amy1A and Amy3D. The relative expression level of *LOC_Os03g48170* increased with a significant difference at the 1% level at 10 days after flowering under high-temperature treatment. The results of BLAST analysis through NCBI showed that LOC_Os03g48170 has a very similar sequence to that of FLOURY ENDOSPERM 6 protein (Fig. 8). The genetic similarity between LOC_Os03g48170 and FLOURY ENDOSPERM 6 present in *Panicum hallii*, *Brachypodium distachyon*, *Zea mays*, and *Setaria viridis* belonging to Gramineae family was confirmed by phylogenetic tree analysis. LOC_Os03g48170 belonged to the same group as the FLOURY ENDOSPERM 6 protein of *B. distachyon* and exhibited the most similar genetic similarities (identity 74%, similarity 80%). Moreover, the genetic similarity to the FLOURY ENDOSPERM 6 protein of *Z. mays* showed the greatest difference (identity 57%, similarity 69%). In addition, as a result of predicting the functional partners using the domain of LOC_Os03g48170, LOC_Os03g48170 was found to interact with 10 different proteins (OS08T0529200–00, OsJ_12316, OS10T0499400–01, OS05T0533600–01, DPE1, OS01T0720600–01, P0453H11.4, OS03T0607500–01, OS01T0180300–01, and OsJ_36662) (Fig. 8).

## Discussion

High-temperature stress is one of the major environmental stresses that can decrease the quality and yield of grains due to negative effects during the development of rice grains [37]. It has been reported to efficiently maintain energy homeostasis to give resistance to various biological and non-biological stresses through the sugar and energy signaling process [38]. This process has been found to play an important role in imparting resistance to disease [39]. During the growing stage of rice, the process of grain development and filling stage is extremely sensitive to temperature changes, and the quality of grains is seriously deteriorated and the yield is reduced due to high-temperature stress [40]. Therefore, in this study, genes that act in the sugar energy signaling transduction process were screened, and genes related to spikelet fertility and grain quality were selected to assess high-temperature stress tolerance during the grain filling stage. High-temperature was administered during the grain filling stage in the CNDH line, and QTL mapping, candidate gene screening, and gene function were predicted and identified using the spikelet fertility and 1000 grain weight that appeared after high-temperature treatment. The CNDH line has been advancing generations in the field of Kyungpook National University every year from 2010 till date [41], and currently, due to the progression of several generations, specific traits are fixed for each line, and it is a bridging parent displaying a wide variety of traits. Therefore, as the expression of the gene is very stable and each line representing a variety of traits is constructed, the amount of expression of the gene involved in a specific trait can be stably confirmed [42]. For QTL mapping of genes involved in spikelet and grain quality under high-temperature treatment during the grain filling stage, we analyzed the content of spikelet fertility and grain quality components (protein, amylose, and moisture) [43–45]. When the phenotype was confirmed after high-temperature treatment in the grain filling stage, chalkiness grains were formed in which the middle portion of the grains turned white [46], and the proportion of perfect grains decreased with a significant difference compared with rice harvested under normal conditions. In the case of grains subjected to high-temperature stress during the grain filling stage, the protein content increased with a significant difference at the level of 1%. In addition, the amylose content decreased with a significant difference at the 1% level, but there was no significant difference in the moisture content under both normal and high-temperature stress conditions. Moreover, high-temperature treatment after flowering also affected the viability of pollen [47]. Under high-temperature treatment, the viability of pollen decreased, which had a direct effect on the spikelet fertility. In particular, the rate of pollen viability decreased in both the high-temperature-sensitive and -tolerant lines, but the rate of decrease in pollen viability was high in the sensitivity line. Exposure to high-temperature during the flowering stage inhibits the elongation of the pollen tube, decreases the number of pollen, and decreases the spikelet fertility by reducing the number of pollen. In addition, even when grains were filling, the weight of grains decreased, and the content of amylose, a component of starch, decreased. The QTLs related to spikelet fertility showed an LOD score of ≥3.0 for 2 consecutive years in RM15749-RM15689 on chromosome 3 and is a commonly searched region. Also RM149-RM23191 on chromosome 8 showed an LOD score of ≥3.0 for 2 consecutive years related to 1000 grain weight. In the QTL mapping analysis, the LOD score was ≥2.5, and it was judged that a relationship existed between the genotype and environment [48, 49], but in this research, the region where the LOD score is 3.0 or higher. RM15749-RM15689 on chromosome 3 and RM149-RM23191 on chromosome 8 contained 43 candidate genes related to spikelet fertility and 1000 grain weight under high-temperature treatment, all of which are genes related to biological processes, molecular functions, and cellular components. Four of these candidate genes (*LOC_Os03g48170*, *LOC_Os03g49600*, *LOC_Os03g49610*, *LOC_Os08g35110*) were involved in functions related to sugar metabolism, and therefore, they were screened as candidate genes related to spikelet fertility and grain quality during the grain filling stage of rice. High-temperature stress during the grain filling stage causes low spikelet fertility by negatively affecting reactive oxygen species (ROS) responses to anther and sugar homeostasis [50]. In the control group, *LOC_Os03g48170*, *LOC_Os03g49600*, *LOC_Os03g49610*, and *LOC_Os08g35110* exhibited no significant difference in their relative expression levels in CNDH11 and CNDH75 at 45 days after flowering. However, there was a significant difference in gene expression levels in CNDH11 and CNDH75 when treated at high-temperature. In particular, *LOC_Os03g48170* showed a difference in expression levels in CNDH11 and CNDH75 at the 1% level after 10 days under high-temperature treatment immediately after the flowering stage. To confirm the effect of high-temperature treatment during the filling stage on the candidate genes and the expression levels of amylose and amylopectin synthase, the expression levels of these genes were also analyzed. The expression levels of the amylose synthesis genes GBSSI [51] and GBSSII were decreased in both CNDH11 and CNDH75, but the rate of decrease in gene expression levels was greater in CNDH11, which was sensitive to high-temperature, than in CNDH75, which was tolerant to high-temperature. Genes responsible for the synthesis of chain elongation of amylopectin SSI [52] and SSIIa did not show a significant difference in CNDH11 and CNDH75, whereas SSIIIa exhibited a significant difference in CNDH11 and CNDH75, and the gene expression level was higher in CNDH11, which was sensitive to high-temperature. Furthermore, when the expression levels of the genes SBEI, SBE11a, and SBEIIb [53] involved in the branching synthesis of amylopectin were analyzed, SBEI exhibited a significant difference at the 1% level after flowering at 10 days under high-temperature treatment and highly expressed in CNDH11. However, when SBE11a and SBE11b were treated at high-temperature, their expression levels were higher than those of control, but there was no significant difference between CNDH11 and CNDH75. In addition, when both the amylose-degrading enzymes Amy1A [54] and Amy3D were subjected to high-temperature treatment, the gene expression levels in CNDH11 showed a significant difference and increased compared to those in CNDH75. Both Amy1A and Amy3D showed a significant difference in expression level at the 1% level in CNDH75 and CNDH11 at 10 days under high-temperature treatment after flowering stage, and after 15 days, Amy1A showed increased expression level in CNDH11 at the 5% level, but Amy3D was 1 in CNDH11. The expression level increased to% level. Starch is composed of amylose and amylopectin, and the absolute amount of starch does not change, but changes in the ratio of amylose and amylopectin affect the grain quality [55]. As shown in Table 1, when subjected to high-temperature, the amylose content decreased more in the high-temperature-sensitive line than in the tolerant line. These results indicate that when the expression levels of the amylopectin synthesis gene, the amylose synthesis gene, and the amylose-degrading enzyme gene were confirmed, the expression of the amylopectin synthesis gene decreased, the expression of the amylopectin synthesis gene increased, and the expression level of the amylose-degrading enzyme gene was increased. This supports the experimental results of increased expression. Among the four candidate genes, we focused on *LOC_Os03g48170*, which showed the greatest rate of increase in the initial reaction under high-temperature treatment and the highest increase in the high-temperature-resistant and -susceptible lines. BLAST analysis of *LOC_Os03g48170* using NCBI revealed that this gene showed a nucleotide sequence similar to that of FLOURY ENDOSPERM 6 protein. *LOC_Os03g48170* showed the highest similarity with FLOURY ENDOSPERM 6 of wheat. FLOURY ENDOSPERM 6 is also involved in seed size, endosperm storage material accumulation, and starch synthesis [56]. In addition, it has been reported that FLOURY ENDOSPERM 6 is involved in heat tolerance during the grain filling stage [57]. When homology is analyzed using their protein sequences, it is predictable that they will have a similar function if they show a high degree of similarity. Moreover, LOC_Os03g48170 interacts with the proteins OS08T0529200–00, OsJ_12316, OS10T0499400–01, OS05T0533600–01, DPE1, OS01T0720600–01, P0453H11.4, OS03T0607500–01, OS01T0180300–01, and OsJ_36662. Among these proteins, OS08T0529200–00 and OS10T0499400–01 are CBS domain-containing proteins. They act as regulators of thioredoxin, one of the antioxidant proteins, and play a vital role in the removal of active oxygen and regulation of developmental processes in plants [58]. OsJ_12316 plays an important role in signaling between cells through plasmodesmata by producing glutamate in plant cells and changing the production of calcium ions [59]. OS05T0533600–01 and OS01T0720600–01 are proteins belonging to the glycosyltransferase 1 family. Glycosyltansferase induces glycosylation in plants and plays a role in storing secondary metabolites and protecting plants from external stresses [60]. Furthermore, glycosyltransferase is essential for intine construction and pollen maturation and plays a vital role in male reproductive development [61]. DPE1 is a protein that encodes 4-alpha-glucanotransferase and does not hydrolyze starch, but it remodels amylose and amylopectin molecules [62]. P0453H11.4, OS03T0607500–01, and OS01T0180300–01 are MAR (matrix attachment region) binding proteins. These are important factors in regulating gene expression through the interaction of chromatin and nucleus. OsJ_36662 is a protein that acts on seed maturation [63].

In this study, QTL mapping of genes that can affect the grain quality and spikelet fertility of rice when subjected to high-temperature treatment during the filling stage of rice and *LOC_Os03g48170* was finally screened from among several candidate genes in RM15749-RM15689 of chromosome 3. The chalkiness appearance of rice due to high-temperature treatment is associated with amylose content, gel consistency, and protein storage [64]. In addition, QTL mapping to reduce the content of chalkiness grain rate under high-temperature conditions, and chalkiness QTL was mapped on chromosome 3 [65]. Also, Ye et al., (2015) [66], QTL related to spikelet fertility was mapped by high-temperature treatment during the flowering stage of rice, and mapping on chromosome 3 using the high-temperature-sensitive variety IR64 and the heat-tolerant variety Giza178. However, Xiao et al., (2011) [67] reported that QTL was detected in chromosomes 4 and 6 under high-temperature treatment during the flowering stage of rice to map the QTL related to spikelet fertility. The reason why QTL was detected in different chromosomes in our study is presumed to be due to genetic factors and environmental differences and the different groups used in the study [68]. In the reproductive stage of rice, high temperatures induce infertility by inducing ROS and sugar homeostasis [69, 70]. After high-temperature treatment during the grain filling stage, the QTLs related to spikelet fertility, chalkiness grain ratio, and sucrose content were analyzed at similar locations on the same chromosome as in this study. QTLs related to spikelet fertility and 1000 grain weight and the major candidate gene *OsSFq3* analyzed under high-temperature treatment during the grain filling stage identified in this study can be used for breeding new rice cultivars that can improve grain quality while being resistant to high temperatures along with the current era of rapid climate change. Furthermore, the linked markers used for QTL mapping can be effectively used for the marker-assisted selection of rice cultivars with high-quality and high-temperature resistance.

## Conclusions

In general, high temperatures cause serious damage by disrupting the sugar signaling system of rice. Rice exposed to high temperatures deteriorates grain quality due to a decrease in amylose content and an increase in protein content. In addition, high temperatures lead to a decrease in yield due to a severe decrease in the rate of spikelet fertility. In this research, four potential candidate genes were screened that could stably maintain grain quality and yield even under high temperatures. Among them, the expression of OsSFq3 increased with a significant difference at the level of 1% of the resistant line compared to the susceptible line from 10 days after high temperature treatment during the flowering of rice. It is very important to give resistance to high temperature in the early stage after rice flowering, and high temperature at the grain filling stage severely negatively affects grain quality. It changes the amylose content and protein content in the grain, and decreases the quality level of all food products made from these grains. In the future, intend to breeding new rice cultivars that are resistant to high temperatures and have good grain quality by development NILs (near-isogenic lines) with OsSFq3 and QTLs detecd in this research. Therefore, OsSFq3 will be effectively used for breeding rice with good taste by resisting high temperatures in the grain filling stage in response to the era of climate change.

## Methods

### Plant material and field design

In this study, Cheongcheong (IT228761, IT number is a resource number managed by the National Academy of Agricultural Sciences of Rural Development Administration, Korea)/Nagdong (IT006182) double haploid line, CNDH, bred through crossing between Cheongcheong (*Oryza sative* spp. *indica* cv. Cheongcheong) and Nagdong (*Oryza sative* spp. *japonica* cv. Nagdong) was used as an experimental material for QTL mapping of spikelet fertility and grain quality genes in rice at high temperatures [41]. Cheongcheong, Nagdong and 120 CNDH lines were obtained from Prof. Kyung-Min Kim at Plant Molecular Breeding laboratory (Kyungpook National University in Korea). In the F_1_ combination formed through crossing between Cheongcheong and Nagdong, 120 CNDH lines that were derived by another culture were bred in the fields of Kyungpook National University (36°6′41.54″N, 128°38′26.17″E) and 120 CNDH lines were cultured in 2019 and 2020. And when conducting research, 120 CNDH line was used in compliance with the international guidelines and legislation provided by RDA (Rural Development Administration) in Korea. Also rice plants were cultivated following normal local practices. This research complied with the Convention on the Trade in Endangered Species of Wild Fauna and Flora (https://www.cites.org/). Before sowing, the seeds were sterilized using a seed disinfectant, and the seeds were soaked by dark treatment at 25 °C for 4 days. On 23 April, 2018, and 25 April, 2019, they were sown in the Kyungpook National University field in Gunwi, South Korea, on May 23, 2019, and May 25, 2020, respectively, and 30 days after sowing, the plants were transplanted. The planting distance was 30 × 15 cm. The amount of fertilization was N-P_2_O_5_-K_2_O = 9–4.5-5.7 kg/10a, which was applied according to the Agricultural Science and Technology Research Survey Standard of RDA. It was cultivated in the field of Kyungpook National University until the flowering stage, and the average was 28 °C during the day time and 22 °C at night time. At 1 day after flowering, the plants were moved to the growth chamber (Vision, VS-8407-1300, Daejeon, Korea) and subjected to high-temperature stress. The high-temperature stress was applied in the growth chamber that was maintained at 42 °C. The light period was 13/11 h (light/dark), the luminous intensity was maintained at 40,000 lx during daytime, and the relative humidity was 70%. Each lines in the 120 CNDH lines has a variety of agricultural traits and a variety of flowering times, so the line where flowering time begins is checked every day and moved to the growth chamber at different times. For high-temperature treatments, the temperature change of the growth chamber was thoroughly controlled over time. Gradually has risen 6:30–7:00 maintained at 27 °C, 7:00–7:30 maintained at 30 °C, 7:30–8:00 maintained at 35 °C, 8:00–8:30 maintained at 38 °C. And 42 °C was maintained for a total of 6 h from 8:30 to 14:30. And it was maintained at 35 °C from 14:30 to 15:30, 30 °C from 15:30 to 16:30, and 27 °C from 16:30 to 17:30. Finally, it was maintained at 25 °C from 17:30 to 6:30. Panicles were sampled immediately after flowering during the process of grain filling and at 5, 10, 15, 20, 35, 30, 35, 40, and 45 days after seeding. Samples were rapidly cooled using liquid nitrogen until they were used in the experiment and then stored at − 80 °C.

### Analysis of scanning electron microscopy for structure of amyloplasts

To observe the grain endosperm, 100 polished rice grains grown under high-temperature and normal conditions were selected. Selected grains were cut using the bladeless part, and the cut cross-section surface was coated with gold in a vacuum using an ion sputtering device (JFC-1100E, JEOL, Tokyo, Japan). And it was observed with a scanning electron microscope with 1.0 KV voltage.

### Pollen viability rate

All anthers were collected from panicles to assess the viability of pollen after the high-temperature stress. Pollen viability is described in Gunawardena et al. (2003) [30]. After high-temperature treatment at the flowering stage, mature pollen were collected from spikelets that have not yet flowering. Anther was stained with 1% iodine potassium iodide solution. For microscopic observation, the stained solution was placed on a glass slide, and the pollen viability was confirmed through the staining state and morphology of anther [71]. When observed under a microscope, the pollen stained in black and round in shape was judged as viable or living, and the pollen stained yellow or light red was judged as inactive or dead. Three biological replicates were performed for each sample in nine areas through the microscope, and pollen viability was evaluated.

### Evaluation of spikelet fertility and 1000 grain weight after high-temperature treatment in grain filling stage

To evaluate the fertility rate of rice after high-temperature treatment, the fertility rate was calculated on the 45th day after flowering. To determine ovary development (filled or not), a fertility test was conducted by pressing every spikelet one by one. All panicles that were completely emptied when pressed were classified as infertile. The spikelet fertility was calculated as the percentage of filled spikelets among the total spikelets. Moreover, the weight was measured using 1000 grains for calculating the grain weight of rice. The number of grains was checked using an auto grain counter (Multi auto counter, WAVER, Japan), and the weight of 1000 grains was measured using an electronic scale (OHAUS, ARD120). To confirm the factors affecting the grain quality at high-temperature, the levels of amylose, protein, and moisture content of seeds were measured by near-infrared spectroscopy (Kett, AN-820, Japan).

### Construction of a genetic map and QTL analysis of heat tolerance in rice

Windows QTL cartographer 2.5 [72] was used for the QTL mapping. Using Mapmaker version 3.0 [73] a genetic map with an average distance between markers of 10.6 cM was generated using 222 SSR markers. The composite interval mapping (CIM) method was used in Kosambi function for the value of the CNDH 120 line, and the accuracy of QTL mapping was improved using a threshold LOD of ≥3.0 [74, 75]. For QTL naming, the method proposed by McCouch, (2008) [76] was used.

### Gene information analysis

After QTL mapping, Rapdb (https://rapdb.dna.affrc.go.jp/) [77] and RiceXpro (https://ricexpro.dna.affrc.go.jp/) [78] were used to select candidate genes for specific traits in the QTL region. These programs present all ORFs (open reading frames) that exist between SSR markers. All ORFs were classified according to their function, and genes related to spikelet fertility and 1000 grain weight at high-temperature were filtered out. The filtered candidate genes were analyzed using the Simple Modular Architecture Research Tool (SMART, http://smart.embl-heidelberg.de/) [79] and ExPASy (https://www.expasy.org) [80] was used to predict the sequence analysis and protein interaction. It also uses NCBI (http://www.ncbi.nim.nih.gov) [81] and BioEdit 7.0 (https://bioedit.software.informer.com/7.0/) [82], so homology multiple sequence can analysis and comparison other homologous genes.

### RNA extraction and PCR protocol

RNA was extracted from Cheongcheong, Nagdong, and CNDH lines using the RNeasy plant mini kit (QIAGEN, Germany). Before RNA extraction, all experimental equipment were cleaned using DEPC, and all experimental procedures were performed at 4 °C to increase the quality and concentration of RNA. Fresh rice panicles were placed in a bowl and ground while freezing the sample using liquid nitrogen. Next, 100 mg of ground rice panicle powder was used for RNA extraction. To 100 mg of powder taken in a 2-ml e-tube, 450 μl of RLT buffer was added and then sufficiently mixed using a vortex. The vortexed solution was added to the QIAshredder spin column and centrifuged at 13,000 rpm for 2 min. The solution filtered under the column was transferred to a new 1.5-ml e-tube, and 0.5 ml of 100% ethanol was added and mixed. The entire mixed solution was placed in the RNeasy mini column and centrifuged at 13,000 rpm for 15 s. Then, 700 μl of RW1 buffer was added and centrifuged at 13,000 rpm for 15 s, followed by addition of 500 μl of RPE buffer and centrifuged at 13,000 rpm. Then, to completely wash the column, 500 μl of RPE buffer was added once again and centrifuged for 2 min at 13,000 rpm. Next, to completely dry the column, it was centrifuged at 13,000 rpm for 2 min without adding any reagent. Finally, 50 μl of RNase-free water was added and centrifuged at 13,000 rpm for 2 min to dissolve the RNA in the column. To assess the quality and concentration of the extracted RNA, it was quantified using an ultramicrospectrophotometer ND-2000 (Nanodrop, USA). cDNA was synthesized using the qPCRBIO cDNA Synthesis Kit (PCRBIOSYSTEMS, USA) according to the manual instructions. RNA 80 ng, 5× cDNA synthesis mix 4 μl, and 20× RTase 1 μl were added, and a final volume of 20 μl was adjusted using RNase-free water. Then, 80 ng of the synthesized cDNA was used as a template for PCR. The PCR solution composition was cDNA 80 ng, 20 pmol forward and reverse primers, 2.5 mM dNTP mixture, Ex Taq polymerase (Inclone Biotech Co., IN5001) 1.0 U, and 3.0 μl of 10× Ex buffer (50 mM KCl, 20 mM Tris-HCl, pH 8.0, 2.0 mM MgCl_2_) in nuclease-free water (QIAGEN, Cat. No. 129114), and it was made a total volume of 30 μl. PCR (C1000, BioRad, USA) conditions were predenaturation at 94 °C for 5 min, denaturation at 94 °C for 30 s, annealing at 55 °C for 30 s, extension at 72 °C for 30 s, and denaturation, annealing, and extension processes repeated for 35 cycles. After that, a final extension step was performed at 72 °C for 5 min, and when the PCR was completed, the products were stored at 4 °C. For sequencing the products amplified through PCR, the sequence was analyzed by SOLGENT Co. The sequencing results were used for homology analysis using the BLAST program of the NCBI (http://www.ncbi.nim.nih.gov) database.

### Analysis of candidate gene expression levels

During the rice flowering stage, a high-temperature of 42 °C was applied to the Cheongcheong, Nagdong, 120 CNDH lines. Furthermore, the panicle of rice was sampled at intervals of 5 days after flowering to check the relative gene expression levels of starch synthase and candidate genes that reacted to high-temperature. Total RNA was extracted from the panicle of rice using the RNeasy plant mini kit (QIAGEN, Germany), and 1 μg of the extracted total RNA was used as a quantitative real-time PCR template for cDNA synthesis and gene expression analysis. cDNA was used as a template for quantitative real-time PCR, which was performed using the Eco Real-Time PCR System. The reaction solution used for quantitative real-time PCR consisted of 2× qRCRBIO SyGreen Blue Mix 10 μl, cDNA 1 μl, forward primer 0.5 μl (20 pmol/μl), and reverse primer 0.5 μl (20 pmol/μl), which was made to a final volume of 20 μl using ddH_2_O (see Additional file 1: Table S3). *OsActin*, a housekeeping gene, was used as a control, and each reaction was performed three times to calculate the mean and standard deviation.

### Statistical analysis

Five repeated experiments were performed for each sample in all control and experimental groups. The mean and standard deviation were calculated using the results through 5 repeated experiments, and the SPSS program (IMMSPSS Statistics, version 22, IBMSPSS Statistics, version 22, Redmond, WC, USA) was used for statistical analysis.

## Supplementary Information

**Additional file 1: Table S1.** QTLs related to the spikelet fertility and 1000 grain weight of the Cheongcheong/Nagdong double haploid population. **Table S2.** Thirty four candidate genes identified between RM15749-RM15689, RM149-RM23191 markers and their ORFs. **Table S3.** Information of the primer sequences used for qPCR.

## Data Availability

The all datasets supporting the conclusions of this article are included in the article and supplementary files. The raw data can be accessed from the NCBI Sequence Read Archive (SRA) platform under the accession number It2455092 LOC_Os03g48170.1 MZ054167 (https://www.ncbi.nlm.nih.gov/sra/).

## References

[1] Wassmann R, Jagadish SVK, Heuer S, Ismail A, Redona E, Serraj R, et al. Chapter 2 climate change affecting rice production. ADV AGRON. 2009;101:59–122. 10.1016/s0065-2113(08)00802-x

[2] Driedonks N, Rieu I, Vriezen WH (2016). Breeding for plant heat tolerance at vegetative and reproductive stages. Plant Reprod.

[3] Jagadish SVK, Raveendran M, Oane R, Wheeler TR, Heuer S, Bennett J (2010). Physiological and proteomic approaches to address heat tolerance during anthesis in rice (Oryza sativa L.). J Exp Bot.

[4] Suzuki M (1980). Studies on distinctive patterens of dry matter production in the building process of grain yields in rice plants grown in the warm region in Japan. Bull Kyushu Nat Agric Exp Sta.

[5] Aghamolki MTK, Yusop MK, Oad FC, Zakikhani H, Jaafar HZ, Kharidah S (2014). Heat stress effects on yield parameters of selected rice cultivars at reproductive growth stages. J Food, Agric Environ.

[6] Chen J, Liang T, Shi P, Yang B, Sun T, Cao W, Zhu Y (2017). Effects of short-term high temperature on grain quality and starch granules of rice (Oryza sativa L.) at post-anthesis stage. Protoplasma..

[7] Liu Q, Wu X, Ma J, Li T, Zhou X, Guo T (2013). Effects of high air temperature on rice grain quality and yield under field condition. Agron J.

[8] IPCC (2014). Synthesis Report.

[9] Peng S, Huang J, Sheehy JE, Laza RC, Visperas RM, Zhong X (2004). Rice yields decline with higher night temperature from global warming. Proc Natl Acad Sci U S A.

[10] Jagadish SVK, Murty MVR, Quick WP (2015). Rice responses to rising temperatures - challenges, perspectives and future directions. Plant Cell Environ.

[11] Jagadish SVK, Craufurd PQ, Wheeler TR (2007). High temperature stress and spikelet fertility in rice (Oryza sativa L.). J Exp Bot.

[12] Wahid A, Gelani S, Ashraf M, Foolad MR (2007). Heat tolerance in plants: an overview. Environ Exp Bot.

[13] Ishimaru T, Miyazaki M, Shigemitsu T, Nakata M, Kuroda M, Kondo M (2020). Effect of high temperature stress during ripening on the accumulation of key storage compounds among Japanese highly palatable rice cultivars. J Cereal Sci.

[14] Yang X, Wang B, Chen L, Li P, Cao C (2019). The different influences of drought stress at the flowering stage on rice physiological traits, grain yield, and quality. Sci Rep.

[15] Wang Y, Wang L, Zhou J, Hu S, Chen H, Xiang J (2019). Research Progress on heat stress of Rice at flowering stage. Rice Sci.

[16] IRRI (1976). Annual Report.

[17] Yoshida S (1981). TS and DSM. High temperature stress in Rice. IRRI research paper series 67.

[18] Yang W, Choi K-J, Shon J, Kang S, Shin S-H, Shim K-B (2015). Effects of temperature and sunshine hours during grain filling stage on the quality-related traits of high quality Rice varieties in Korea. Korean J Crop Sci.

[19] Xie L, Tang S, Chen N, Luo J, Jiao G, Shao G (2013). Rice grain morphological characteristics correlate with grain weight and milling quality. Cereal Chem.

[20] Bian JM, Shi H, Li CJ, Zhu CL, Yu QY, Peng XS (2013). QTL mapping and correlation analysis for 1000-grain weight and percentage of grains with chalkiness in rice. J Genet.

[21] Chun A, Song J, Kim K-J, Lee H-J (2009). Quality of head and chalky rice and deterioration of eating quality by chalky rice. J Crop Sci Biotechnol.

[22] Smeekens S, Ma J, Hanson J, Rolland F (2010). Sugar signals and molecular networks controlling plant growth. Curr Opin Plant Biol.

[23] Zhang CX, Feng BH, Chen TT, Fu WM, Li HB, Li GY (2018). Heat stress-reduced kernel weight in rice at anthesis is associated with impaired source-sink relationship and sugars allocation. Environ Exp Bot.

[24] Yaliang W, Yikai Z, Qinghua S, Huizhe C, Jing X, Guohui H (2020). Decrement of sugar consumption in Rice young panicle under high temperature aggravates spikelet number reduction. Rice Sci.

[25] Sami F, Yusuf M, Faizan M, Faraz A, Hayat S (2016). Role of sugars under abiotic stress. Plant Physiol Biochem.

[26] Elsayed AI, Rafudeen MS, Golldack D (2014). Physiological aspects of raffinose family oligosaccharides in plants: protection against abiotic stress. Plant Biol.

[27] Maruyama A, Weerakoon WMW, Wakiyama Y, Ohba K (2013). Effects of increasing temperatures on spikelet fertility in different Rice cultivars based on temperature gradient chamber experiments. J Agron Crop Sci.

[28] Das S, Krishnan P, Nayak M, Ramakrishnan B (2014). High temperature stress effects on pollens of rice (Oryza sativa L.) genotypes. Environ Exp Bot.

[29] Prasad PVV, Boote KJ, Allen LH, Sheehy JE, Thomas JMG (2006). Species, ecotype and cultivar differences in spikelet fertility and harvest index of rice in response to high temperature stress. F Crop Res.

[30] Fahad S, Ihsan MZ, Khaliq A (2018). Consequences of high temperature under changing climate optima for rice pollen characteristics-concepts and perspectives. Arch Agron Soil Sci.

[31] Endo M, Tsuchiya T, Hamada K, Kawamura S, Yano K, Ohshima M (2009). High temperatures cause male sterility in rice plants with transcriptional alterations during pollen development. Plant Cell Physiol.

[32] Boden SA, Kavanová M, Finnegan EJ, Wigge PA (2013). Thermal stress effects on grain yield in Brachypodium distachyon occur via H2A.Z-nucleosomes. Genome Biol.

[33] Jansen RC, Van Ooijen JW, Stam P, Lister C, Dean C (1995). Genotype-by-environment interaction in genetic mapping of multiple quantitative trait loci. Theor Appl Genet.

[34] El-Soda M, Malosetti M, Zwaan BJ, Koornneef M, Aarts MGM (2014). Genotype × environment interaction QTL mapping in plants: lessons from Arabidopsis. Trends Plant Sci.

[35] Nadeau JH, Frankel WN (2000). The roads from phenotypic variation to gene discovery: mutagenesis versus QTLs. Nat Genet.

[36] Boddhireddy P, Jannink JL, Nelson JC (2009). Selective advance for accelerated development of recombinant inbred QTL mapping populations. Crop Sci.

[37] Ahmed N, Tetlow IJ, Nawaz S, Iqbal A, Mubin M (2015). Nawaz ul Rehman MS, et al. effect of high temperature on grain filling period, yield, amylose content and activity of starch biosynthesis enzymes in endosperm of basmati rice. J Sci Food Agric.

[38] Gong X, Liu M, Zhang L, Ruan Y, Ding R, Ji Y (2015). Arabidopsis AtSUC2 and AtSUC4, encoding sucrose transporters, are required for abiotic stress tolerance in an ABA-dependent pathway. Physiol Plant.

[39] Van den Ende W, El-Esawe SK (2014). Sucrose signaling pathways leading to fructan and anthocyanin accumulation: a dual function in abiotic and biotic stress responses?. Environ Exp Bot.

[40] Cheng C, Ali A, Shi Q, Zeng Y, Tan X, Shang Q (2018). Response of chalkiness in high-quality rice (Oryza sativa L.) to temperature across different ecological regions. J Cereal Sci.

[41] Lee GH, Yun BW, Kim KM (2014). Analysis of QTLs associated with the rice quality related gene by double haploid populations. Int J Genomics.

[42] Park JR, Yang WT, Kim DH, Kim KM (2020). Identification of a novel gene, osbht, in response to high temperature tolerance at booting stage in rice. Int J Mol Sci.

[43] Perez CM, Juliano BO, Liboon SP, Alcantara JM, Cassman KG (1996). Effects of late nitrogen fertilizer application on head rice yield, protein content, and grain quality of rice. Cereal Chem.

[44] Bhattacharya KR, Sowbhagya CM, Indudhara Swamy YM (1978). Importance of insoluble amylose as a determinant of rice quality. J Sci Food Agric.

[45] Zheng X, Lan Y (2007). Effects of drying temperature and moisture content on rice taste quality.

[46] Nakata M, Fukamatsu Y, Miyashita T, Hakata M, Kimura R, Nakata Y (2017). High temperature-induced expression of rice α-amylases in developing endosperm produces chalky grains. Front Plant Sci.

[47] Kumar N, Kumar N, Shukla A, Shankhdhar SC, Shankhdhar D (2015). Impact of terminal heat stress on pollen viability and yield attributes of rice (Oryza sativa L.). Cereal Res Commun.

[48] Churchill GA, Doerge RW (1994). Empirical threshold values for quantitative trait mapping. Genetics..

[49] Crump T, Llewellyn-Thomas HA (2011). Assessing medicare beneficiaries’ strength-of-preference scores for health care options: how engaging does the elicitation technique need to be?. Health Expect.

[50] Rezaul IM, Baohua F, Tingting C, Weimeng F, Caixia Z, Longxing T (2019). Abscisic acid prevents pollen abortion under high-temperature stress by mediating sugar metabolism in rice spikelets. Physiol Plant.

[51] Edwards A, Vincken JP, Suurs LCJM, Visser RGF, Zeeman S, Smith A (2002). Discrete forms of amylose are synthesized by isoforms of GBSSI in pea. Plant Cell.

[52] Delvallé D, Dumez S, Wattebled F, Roldán I, Planchot V, Berbezy P (2005). Soluble starch synthase I: a major determinant for the synthesis of amylopectin in Arabidopsis thaliana leaves. Plant J.

[53] Brummell DA, Watson LM, Zhou J, McKenzie MJ, Hallett IC, Simmons L (2015). Overexpression of STARCH BRANCHING ENZYME II increases short-chain branching of amylopectin and alters the physicochemical properties of starch from potato tuber. BMC Biotechnol.

[54] Hakata M, Kuroda M, Miyashita T, Yamaguchi T, Kojima M, Sakakibara H (2012). Suppression of α-amylase genes improves quality of rice grain ripened under high temperature. Plant Biotechnol J.

[55] Fredriksson H, Silverio J, Andersson R, Eliasson AC, Åman P (1998). The influence of amylose and amylopectin characteristics on gelatinization and retrogradation properties of different starches. Carbohydr Polym.

[56] Peng C, Wang Y, Liu F, Ren Y, Zhou K, Lv J (2014). FLOURY ENDOSPERM6 encodes a CBM48 domain-containing protein involved in compound granule formation and starch synthesis in rice ENDOSPERM. Plant J.

[57] She KC, Kusano H, Koizumi K, Yamakawa H, Hakata M, Imamura T (2010). A novel factor FLOURY ENDOSPERM2 is involved in regulation of rice grain size and starch quality. Plant Cell.

[58] Jeong BC, Park SH, Yoo KS, Shin JS, Song HK (2013). Crystal structure of the single cystathionine β-synthase domain-containing protein CBSX1 from Arabidopsis thaliana. Biochem Biophys Res Commun.

[59] Toyota M, Spencer D, Sawai-toyota S, Jiaqi W, Zhang T (2018). R ES E A RC H.

[60] Ko JH, Kim BG, Hur HG, Lim Y, Ahn JH (2006). Molecular cloning, expression and characterization of a glycosyltransferase from rice. Plant Cell Rep.

[61] Moon S, Kim SR, Zhao G, Yi J, Yoo Y, Jin P (2013). Rice GLYCOSYLTRANSFERASE1 encodes a GLYCOSYLTRANSFERASE essential for pollen wall formation. Plant Physiol.

[62] Van Der Maarel MJEC, Leemhuis H (2013). Starch modification with microbial alpha-glucanotransferase enzymes. Carbohydr Polym.

[63] Harder PA, Silverstein RA, Meier I (2000). Conservation of matrix attachment region-binding filament-like protein 1 among higher plants. Plant Physiol.

[64] Nevame AYM, Emon RM, Malek MA, Hasan MM, Amirul Alam M, Muharam FM (2018). Relationship between high temperature and formation of chalkiness and their effects on quality of rice. Biomed Res Int.

[65] Wada T, Miyahara K, Sonoda JY, Tsukaguchi T, Miyazaki M, Tsubone M (2015). Detection of QTLs for white-back and basal-white grains caused by high temperature during ripening period in japonica rice. Breed Sci.

[66] Ye C, Tenorio FA, Argayoso MA, Laza MA, Koh HJ, Redoña ED (2015). Identifying and confirming quantitative trait loci associated with heat tolerance at flowering stage in different rice populations. BMC Genet.

[67] Xiao YH, Pan Y, Luo LH, Deng HB, Zhang GL, Tang WB (2011). Quantitative trait loci associated with pollen fertility under high temperature stress at flowering stage in rice (*Oryza sativa*). Rice Sci.

[68] Xiao J, Li J, Yuan L, Tanksley SD (1996). Identification of QTLs affecting traits of agronomic importance in a recombinant inbred population derived from a subspecific rice cross. Theor Appl Genet.

[69] Parrotta L, Faleri C, Cresti M, Cai G (2016). Heat stress affects the cytoskeleton and the delivery of sucrose synthase in tobacco pollen tubes. Planta..

[70] Zhou Z, Yuan Y, Zhou W, Zhang C (2016). Effects of exogenously supplied sucrose on OsSUTs and OsSPSs transcript abundances and rice root ammonium assimilation. Acta Physiol Plant.

[71] Li HS (2000). Experimental principle and technology of plant physiology and biochemistry.

[72] Wang S (2012). Basten and ZBZ. Windows QTL cartographer 2.5.

[73] Lincoln S (1992). Construcing genetic maps with MAPMAKER/EXP 3.0. Whitehead Institute Technical Report.

[74] Zeng ZB (1993). Theoretical basis for separation of multiple linked gene effects in mapping quantitative trait loci. Proc Natl Acad Sci U S A.

[75] Zeng ZB (1994). Precision mapping of quantitative trait loci. Genetics..

[76] McCouch SR (2008). Gene nomenclature system for rice. Rice..

[77] Sakai H, Lee SS, Tanaka T, Numa H, Kim J, Kawahara Y (2013). Rice annotation project database (RAP-DB): an integrative and interactive database for rice genomics. Plant Cell Physiol.

[78] Sato Y, Takehisa H, Kamatsuki K, Minami H, Namiki N, Ikawa H (2013). RiceXPro version 3.0: expanding the informatics resource for rice transcriptome. Nucleic Acids Res.

[79] Letunic I, Copley RR, Pils B, Pinkert S, Schultz J, Bork P (2006). SMART 5: domains in the context of genomes and networks. Nucleic Acids Res.

[80] Cash P (1999). 2-D proteome analysis protocols. Cell Biol Int.

[81] Pruitt KD, Tatusova T, Maglott DR (2007). NCBI reference sequences (RefSeq): a curated non-redundant sequence database of genomes, transcripts and proteins. Nucleic Acids Res.

[82] Hall T (2004). Bioedit 7.0. 0.

