# Supplementary material for: Screening and identification of genes affecting grain quality and spikelet fertility during high-temperature treatment in grain filling stage of rice
Source: BMC Plant Biol. 2021 Jun 7;21:263. doi: 10.1186/s12870-021-03056-9 (PMC8186072; doi:10.1186/s12870-021-03056-9)
Supplement: Supplementary file 1 — Additional file 1: Table S1. QTLs related to the spikelet fertility and 1000 grain weight of the Cheongcheong/Nagdong double haploid population. Table S2. Thirty four candidate genes identified between RM15749-RM15689, RM149-RM23191 markers and their ORFs. Table S3. Information of the primer sequences used for qPCR. [file 12870_2021_3056_MOESM1_ESM.docx]

Supporting Information

**Screening and identification of genes affecting grain quality and spikelet fertility during high-temperature treatment in grain filling stage of rice**

Jae-Ryoung Park^1,2^, Eun-Gyeong Kim^1^, Yoon-Hee Jang^1^ and Kyung-Min Kim^1,2*^

^1^ Division of Plant Biosciences, School of Applied Biosciences, College of Agriculture and Life Science, Kyungpook National University, Daegu 41566, Republic of Korea. ^2^ Coastal Agriculture Research Institute, Kyungpook National University, Daegu 41566, Republic of Korea.

*Corresponding author: Kyung-Min Kim Ph.D., School of Applied Biosciences, College of Agriculture & Life Sciences, Kyungpook National University, Daegu, 41566, Korea. (Phone) +82-53-950-5711; (E-mail) kkm@knu.ac.kr

**Additional file**

**Table S1.** QTLs related to the spikelet fertility and 1,000 grain weight of the Cheongcheong/Nagdong double haploid population.

| Characteristics | Year | QTL | Chromosome | Interval Markers^z^ | LOD | Additive effect ^y^ | *R^2^*^x^ | Increasing effects ^w^ |
| --- | --- | --- | --- | --- | --- | --- | --- | --- |
| Spikelet fertility (%) | 2019 | qSf3 | 3 | RM15749-RM2334 | 3.2 | 0.3 | 0.2 | Cheongcheong |
|  |  | qSf4 | 4 | RM1205-RM3330 | 2.8 | 0.4 | 0.1 | Cheongcheong |
|  | 2020 | qSf3-1 | 3 | RM6266-RM15689 | 5.4 | 0.7 | 0.1 | Cheongcheong |
|  |  | qSf8 | 8 | RM264-RM23581 | 3.1 | 0.3 | 0.3 | Cheongcheong |
| 1000 grain weight (g) | 2019 | qTgw8 | 8 | RM23178-RM23191 | 2.9 | -1.3 | 0.2 | Nagdong |
|  | 2020 | qTgw7 | 7 | RM248-RM1134 | 2.7 | 3.2 | 0.2 | Cheongcheong |
|  |  | qTgw8-1 | 8 | RM149-RM23191 | 3.4 | -0.2 | 0.1 | Nagdong |

Sf, Spikelet fertility; Tgw, 1,000-grain weight

^z^ Interval markers are those within the significance threshold on each border of the QTL range

^y^ Positive values of the additive effect indicate that alleles from Cheongcheong are in the direction of increasing the traits

^x^ The proportion of evaluated phenotype variation attributable to a particular QTL was estimated by the coefficient of determination (*R^2^*)

^w^ Increase allele is the source of the allele causing an increase in the measured trait

**Table S2.** Thirty four candidate genes identified between RM15749-RM15689, RM149-RM23191 markers and their ORFs.

| Chromosome | Marker interval | Locus | Description |
| --- | --- | --- | --- |
| 3 | RM15749-RM15689 | Os03g0686900 | Glycoside hydrolase, family 13, N-terminal domain containing protein. |
|  |  | Os03g0692000 | Glycosyl transferase, family 14 protein. |
|  |  | Os03g0692500 | Galectin, galactose-binding lectin family protein. |
|  |  | Os03g0693600 | Similar to Indole-3-acetate beta-glucosyltransferase (EC 2.4.1.121) (IAA-Glu synthetase). |
|  |  | Os03g0693700 | Similar to Oxalate oxidase 1 (EC 1.2.3.4) (Germin). |
|  |  | Os03g0693800 | Similar to Oxalate oxidase 1 (EC 1.2.3.4) (Germin). |
|  |  | Os03g0693900 | Similar to Oxalate oxidase 1 (EC 1.2.3.4) (Germin). |
|  |  | Os03g0694000 | Similar to Oxalate oxidase 1 (EC 1.2.3.4) (Germin). |
|  |  | Os03g0695600 | Proteasome subunit beta type 2 (EC 3.4.25.1) (20S proteasome alpha subunit D). |
|  |  | Os03g0695700 | Similar to Chloride channel protein CLC-d (AtCLC-d). |
|  |  | Os03g0696300 | CCAAT-binding transcription factor, subunit B family protein. |
|  |  | Os03g0698800 | Zinc finger, CCCH-type domain containing protein. |
|  |  | Os03g0698900 | Alkaline phytoceramidase family protein. |
|  |  | Os03g0701200 | Similar to Sugar-starvation induced protein (Fragment). |
|  |  | Os03g0702000 | UDP-glucuronosyl/UDP-glucosyltransferase family protein. |
|  |  | Os03g0702500 | UDP-glucuronosyl/UDP-glucosyltransferase family protein. |
|  |  | Os03g0703000 | Similar to Beta-glucosidase. |
|  |  | Os03g0703100 | Similar to Beta-glucosidase. |
|  |  | Os03g0703200 | Protein kinase-like domain containing protein. |
|  |  | Os03g0704700 | Oxysterol-binding protein family protein. |
|  |  | Os03g0706900 | Zinc finger, RING-type domain containing protein. |
|  |  | Os03g0707600 | OsGAI. |
|  |  | Os03g0708100 | Phytanoyl-CoA dioxygenase family protein. |
|  |  | Os03g0708900 | Zinc finger, RanBP2-type domain containing protein. |
|  |  | Os03g0710100 | Protein kinase-like domain containing protein. |
|  |  | Os03g0710500 | Similar to Luminal binding protein 2 precursor (BiP2) (Heat shock protein 70 homolog 2). |
| 8 | RM149-RM23191 | Os08g0439000 | Phosphofructokinase family protein. |
|  |  | Os08g0439900 | Mitochondrial glycoprotein family protein. |
|  |  | Os08g0440100 | Similar to Temperature stress-induced lipocalin. |
|  |  | Os08g0442300 | Similar to Calcineurin-like protein. |
|  |  | Os08g0452500 | Auxin responsive SAUR protein family protein. |
|  |  | Os08g0452900 | Non-protein coding transcript, unclassifiable transcript. |
|  |  | Os08g0453200 | Dormancyauxin associated family protein. |
|  |  | Os08g0459700 | Similar to Adenosine diphosphate glucose pyrophosphatase precursor. |

**Table S3.** Information of the primer sequences used for qPCR.

| Gene | primer | DNA sequence (5’ to 3’) | Annealing temperature (°C) | Predicted PCR product size (bp) |
| --- | --- | --- | --- | --- |
| *OsActin* | Forward | TGTTTTCTGTTCGCTGCAAG | 60 | 80 |
|  | Reverse | CAAACCCTCCAGGAAATCAA | 59 |  |
| *OsSFq3* | Forward | CGACGATTGGGAATTTGAAG | 60 | 76 |
|  | Reverse | TGCTATTTTTGCACGGATTG | 59 |  |
| *LOC_Os03g49610* | Forward | AAATGGCGGATCTATTCACG | 59 | 72 |
|  | Reverse | ACCAGTGCTTGACACGATTG | 59 |  |
| *LOC_Os03g0703100* | Forward | CACATCAAAGTTCGGCATTG | 60 | 74 |
|  | Reverse | ACCAGTTGGCTGAGTCCTTG | 60 |  |
| *LOC_Os08g35110* | Forward | GTGTACGTCGGCAAGTCG | 58 | 79 |
|  | Reverse | CGACGAGGTTCTGGAACAG | 59 |  |
| *GBSSI* | Forward | ACTGGCGAGCTACCTGAAGA | 60 | 79 |
|  | Reverse | GTTGTGGATGCAGAAAGCAA | 59 |  |
| *GBSSII* | Forward | CTTTCAGGTATGGGGCAAGA | 60 | 76 |
|  | Reverse | CAACTGGTTGTCCCGGTAGT | 59 |  |
| *SSI* | Forward | GTAGTGTTCACTCCGCAGCA | 60 | 73 |
|  | Reverse | CCCAAAATGCAGGTATGCTT | 59 |  |
| *SSIIa* | Forward | CGAGACACCGTTGAGCACTA | 60 | 78 |
|  | Reverse | TCTGTTTGCCTCTGCCTTTT | 59 |  |
| *SSIIIa* | Forward | GAAGAATCATCGGAGGTGGA | 60 | 70 |
|  | Reverse | CATCATCCATTCCCTCCAAG | 59 |  |
| *SBEI* | Forward | ACATGATGGATCAGCAGCAC | 60 | 73 |
|  | Reverse | CTTGTCGTCAGAGCCTCCTC | 59 |  |
| *SBEIIa* | Forward | CCAATGATTCCTTGCGTTTT | 60 | 73 |
|  | Reverse | AACTCCGTGGTGTTTCATCC | 59 |  |
| *SBEIIb* | Forward | CCAGTTTCCTGGCTTTACCA | 60 | 75 |
|  | Reverse | AATCCATACATGCACCGTGA | 59 |  |
| *Amy1A* | Forward | CAGGTCGTTGAATTGCTTGA | 60 | 73 |
|  | Reverse | CTTAGACGCGTCCAGATCGT | 59 |  |
| *Amy3D* | Forward | ATTTCGAGGTCCGGATTAGG | 60 | 72 |
|  | Reverse | TCGTCCTCCTTCAATTCCTG | 59 |  |
